# Electrical parameters extraction of PV modules using artificial hummingbird optimizer

**DOI:** 10.1038/s41598-023-36284-0

**Published:** 2023-06-07

**Authors:** Ragab El-Sehiemy, Abdullah Shaheen, Attia El-Fergany, Ahmed Ginidi

**Affiliations:** 1 Department of Electrical Engineering, Faculty of Engineering, Kafrelsheikh University, Kafrelsheikh, 33516 Egypt; 2 Department of Electrical Engineering, Faculty of Engineering, Suez University, Suez, 43533 Egypt; 3 Electrical Power and Machines Department, Faculty of Engineering, Zagazig University, Zagazig, 44519 Egypt

**Keywords:** Energy science and technology, Engineering, Mathematics and computing

## Abstract

The parameter extraction of PV models is a nonlinear and multi-model optimization problem. However, it is essential to correctly estimate the parameters of the PV units due to their impact on the PV system efficiency in terms of power and current production. As a result, this study introduces a developed Artificial Hummingbird Technique (AHT) to generate the best values of the ungiven parameters of these PV units. The AHT mimics hummingbirds' unique flying abilities and foraging methods in the wild. The AHT is compared with numerous recent inspired techniques which are tuna swarm optimizer, African vulture’s optimizer, teaching learning studying-based optimizer and other recent optimization techniques. The statistical studies and experimental findings show that AHT outperforms other methods in extracting the parameters of various PV models of STM6-40/36, KC200GT and PWP 201 polycrystalline. The AHT’s performance is evaluated using the datasheet provided by the manufacturer. To highlight the AHT dominance, its performance is compared to those of other competing techniques. The simulation outcomes demonstrate that the AHT algorithm features a quick processing time and steadily convergence in consort with keeping an elevated level of accuracy in the offered solution.

## Introduction

Solar energy is a promising renewable technology due to its environmental responsiveness and numerous supplies. Solar Photovoltaic (PV) system development is continuing, which encourages the effective use of these systems in generating electric power to meet the need for energy^1^. Also, there are several drawbacks to the performance of PV systems, such as insufficient PV panel productivity and direct panel disclosure to the elements^2^. As a result, determining the realistic efficiency of PV systems is critical for efficiently planning, controlling, and simulating PV modules. To achieve this goal, the practical model is employed based on the current and voltage samples that are gathered at the module terminals. PV parameters may be established, and its model can be built with the aid of mathematical representation.

In the literature, many researchers have developed a variety of PV models, including the Single-Diode Model (SDM) and Double-Diode Model (DDM). Furthermore, PV model performance depends on unidentified internal parameters. Due to degradation, aging, and unpredictable functioning states, keeping all the unknown parameters steady and evaluating them is challenging. Designing, estimating, simulating, and optimizing PV modules is impossible without establishing their regarding electrical parameters. As a result, the effectiveness of swarm optimizing methods for quantifying PV system parameters is being studied^3^. Analytical approaches^4^ create simplified assumptions or particular approximations with ignoring compromising accuracy. However, this analytical model has been simplified by ignoring the effect of parallel and series resistances in calculating the current and voltage related to the highest power output. In^5^, Lagrange Multiplier Method (LMM) have been proposed for SDM/DDM to optimize power outputs of solar cell PV modules. In^6^, the crucial information were reduced from the datasheet of manufacturer where a bounding requirement for a zero-voltage state has been created using the power first derivative. Furthermore, in^7^, four random locations have been illustrated on the I–V curve and their slopes to extract the SDM parameters analytically without approximation or simplification. However, such analytical approach is limited to conventional testing scenarios. Which has a lot of calculations and fails when they change^8^.

On the other side, numerical approaches including deterministic and metaheuristic algorithms have been presented. Inaccurate initial values might lead to local optima in the deterministic method as well as the real model finds it challenging to satisfy the objective function equation's limitations^9^. Conversely, metaheuristic methods provide an effective and simple approach of determining PV model parameters. As a result, the parameters extraction topic has been addressed with a study of metaheuristic methods. Myriads of research have conducted using Differential Evolution (DE)^10,11^ to address the parameter identification issue with the PV model. In^12^, a comparable DE containing a reversed learning process, multi-population strategies, and mutation strategy have been proposed for SDM and DDM. To predict the PV cell characteristics of the RTC France solar cell and the Photowatt-PWM201, two straightforward, metaphor-free methods, Rao-2 and Rao-3, have been performed^13^. Gorilla troops optimizer^14^, slime mould optimization algorithm^15^, Improved Grasshopper Optimization Algorithm (IGOA)^16^ have been developed for electrically solar PV systems. In^17^, Harris hawks optimization was integrated with the Nelder-Mead simplex and (horizontal and vertical) crossovers and implemented to KC200GT, SM55, and ST40 including DDM and SDM. Furthermore, JAYA algorithm has been modified using the chaotic map in^18^ and combined with a learning strategy based on elite opponent process in^19^ to extract the PV parameter extraction.

A DE was integrated with Teaching–Learning Based Optimizer (TLBO) in^20^ taking into consideration the learners’ ranking probability. In^21^, Gradient-based optimization (GBO) with a logistic map and rank-based strategy has been elaborated to both SM55, KC200GT with the SDM and DDM. In^22^, an innovative two-stage technique for extracting the parameters of the PV module's SDM from experimental data of Power-Voltage characteristics. In ref.^23^, an elite strategy was incorporated with backtracking search approach (BSA) which implemented for both SDM and DDM. In^24^, coot-bird optimizer was applied for estimating the optimal PV parameters considering different electrical models based on the number of diode branches. In^25^, an improved TLBO (ITLBO) was emerged with different teaching tactics and performed in a comparative way on both SDM and DDM. In^26^, a grey Wolf algorithm having an orthogonal learning strategy has been manifested for finding the unknowns of different solar PV models. Considering the Triple-Junction (TJS) PV panel, moth search^27^, water cycle^28^ and heap optimizer^29^ techniques were applied for extracting the parameters of InGaP/InGaAs/Ge TJS PV panel. In^27–29^, when operating a TJSC-based PV module at varied irradiances and temperatures, their performances were assessed and confirmed against several other optimizing strategies.

It is established by a variety of "no free lunch" (NFL) theories that every algorithm's improved performance over one class of problems is counterbalanced by efficiency over another^30^. Numerous alternative metaheuristics were illustrated for optimally identifying the unknown parameters of PV models. The CPU time available, constraints, and some observations regarding these methods are also shown in the literature. The proposed Artificial hummingbird technique AHT^31^ by (Zhao et al.) is implemented in this paper to overcome the disadvantages of other techniques. The AHT imitates the special flying and foraging patterns of hummingbirds. The implementation of territorial, directed and migratory foraging’s, as well as the construction of visiting tables can mimic the memorization capacity of these birds for foods This article's main contributions can be summed up in the following manner: (i) AHT demonstrates successfully the PV model parameters, (ii) The results attained from the AHT are compared with new recently developed techniques which are Tuna swarm optimizer (TSO)^32^, African vultures optimizer (AVO)^33^, Teaching learning studying-based optimizer (TLSBO)^34^, (iii) Diverse SDM and DDM PV model designs are used to compare AHT to other well-known reporting approaches, and (iv) Statistical tests and experimental results demonstrate the viability of the AHT.

The remaining items are grouped as follows. Diverse solar cell models are presented “Problem formulation for SDM and DDM” section, while “AHT for extraction of PV cell parameters” section manifests the proposed AHT for PV parameters extraction. Statistical analyses and experimental findings of several PV models are illustrated in "Simulation results" section. The suggested work's conclusions are shown and underlined in "Conclusion" section.

## Problem formulation for SDM and DDM

This part illustrates the non-linear P–V and I–V characteristics of numerous photovoltaic model’s solar cell. additionally, the most common PV model designs will be described and the corresponding electric circuits and the mathematical equations for these PV models will be described^35^.

### SDM

**Figure 1:**
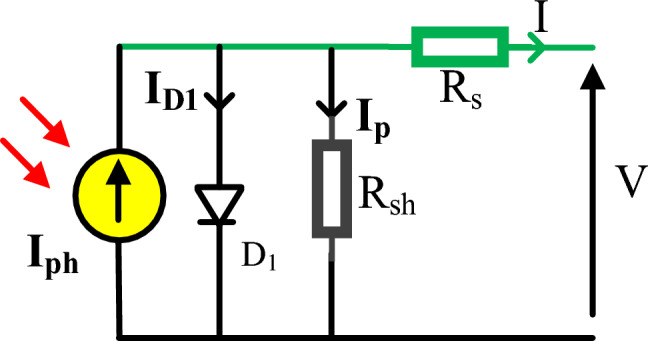
Equivalent circuit for SDM.

This configuration typically contains of one diode (D) in parallel with shunt resistor ($R_{{{\text{sh}}}}$) and photo-generated current ($I_{{{\text{ph}}}}$). This configuration, as manifested in Fig. 1, is put in series with another ($R_{{\text{S}}}$). The SDM current output (I) is mathematically formulate^36,37^:

1 $$I = I_{ph} - I_{SD1} \left[ {\exp \left( {\frac{{V + IR_{S} }}{{n_{1} .V_{tm} }}} \right) - 1} \right] - \frac{{V + IR_{S} }}{{R_{sh} }}$$

2 $$V_{tm} = \frac{{K_{b} T}}{q}$$

where $I_{P}$, $I_{D1}$, and $I_{SD1}$ denote the current through shunt resistor, diode current, the diode's reverse saturating current, accordingly. Moreover, the symbol ($n_{1}$*)* characterizes its ideality factor, while the symbol (*V*) demonstrates the output voltage. In addition to that the symbol ($V_{tm}$) indicates the thermal voltage, whereas the electron charge (*q*) value is *C*, the Boltzmann constant ($K_{b}$) value is J/K, and *T* refers to the cell temperature. In this context, it is important to define five parameters *f*($I_{ph}$, $R_{S}$, $n$*,* $I_{SD}$, $R_{sh}$) accurately from (1).

### DDM

**Figure 2:**
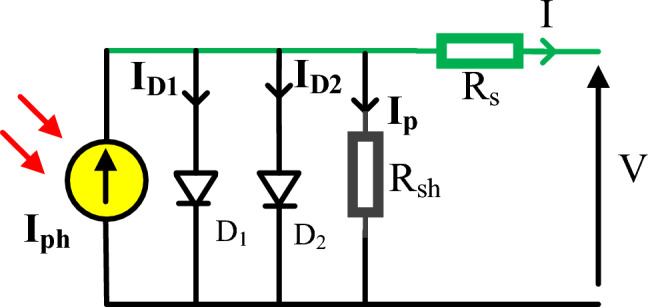
Equivalent circuit for DDM.

It is considered as a SDM with insertion of an additional recombination diode (D2) as manifested with aid of Fig. 2. Although SDM has advantages of its simple structure, fewer parameters to extract, and rapid implementation in the depletion area, It ignores the recombination loss at low voltage which is pivotal when using the practical solar cell^38,39^. Accordingly, Although the structure of SDM is less difficult than DDM, DDM gets higher performance. The DDM output current (I) is mathematically represented by:

3 $$I = I_{Ph} - I_{SD1} \left[ {\exp \left( {\frac{{V + IR_{S} }}{{n_{1} V_{th} }}} \right) - 1} \right] - I_{SD2} \left[ {\exp \left( {\frac{{V + IR_{S} }}{{n_{2} V_{th} }}} \right) - 1} \right] - \frac{{V + IR_{S} }}{{R_{sh} }}$$

where ${\text{I}}_{{{\text{SD}}1}}$ illustrates diffusion current regarding the first diode, while ${\text{I}}_{{{\text{SD}}2}}$ demonstrates saturation current regarding the second diode. Besides, ${\text{I}}_{{{\text{D}}2}}$. and ${\text{n}}_{2}$ reveal the current of diffusion and recombination diodes and its quality factor. Using (3), compared to SDM, there are now seven additional parameters to be determined e.g. $\left( {{\text{I}}_{{{\text{Ph}}}} ,{\text{ R}}_{{\text{S}}} ,{\text{ I}}_{{{\text{SD}}1}} ,{\text{n}}_{1} ,{\text{ I}}_{{{\text{SD}}2}} ,{\text{n}}_{2} ,{\text{ R}}_{{{\text{sh}}}} } \right)$.

### Objective Function

The objective function can diminish the error among simulated and experimental current by defining the optimal estimation of the electrical parameters of the two models (SDM and DDM). The root mean square error (RMSE)^40^ is used as an objective function to determine the change between two *I*–*V* characteristics. It can be illustrated as follows:

4a $$RMSE = \sqrt {\frac{1}{M}\mathop \sum \limits_{1}^{M} \left( {I_{\exp }^{j} - I_{cal}^{j} \left( {V_{\exp }^{j} , x} \right)} \right)^{2} }$$

where *M* explains the number of experimental data points; $I_{exp}^{j}$ and $V_{exp}^{j}$ represent the current and voltage values of *j*^*th*^ experimental point, respectively; $I_{cal}^{j} \left( {V_{exp}^{j} , x} \right)$ denotes the computed current output; and the variable *x* implies the decision parameters.

The objective presented in Eq. (4a) is so traditional and implemented by various reported works. Based on this objective model, the main focus is for minimizing the error aggregation, but it doesn’t guarantee the direction for minimizing the maximum error which may be produced via a single experimental recording. Despite the objective model in Eq. (4a) provides significant coincidence for the whole PV characteristics, the error in some readings would be high. Therefore, another objective function is dedicated to minimize the summation of current absolute error (MAE) over the number of experimental data points which can be mathematically modeled as follows:

4b $$OF = Min\left( \sum \limits_{1}^{M}{\left| {I_{\exp }^{j} - I_{cal}^{j} \left( {V_{\exp }^{j} , x} \right)} \right|} \right),\quad j = 1,2, \ldots .M$$

Using the proposed objective in Eq. (4b), the searching direction is dedicated for minimizing the maximum error over the course of the experimental recordings and so the distribution of errors will be approximately equivalent and more suitable.

## AHT for extraction of PV cell parameters

In the AHT’s procedures, each hummingbird is assigned a definite food supply from which it can be feed. For this specific feed supply, it can memorize the rate and location of nectar replenishment. Moreover, it remembers how often it has been since it last accessed every source of food. The AHT has exceptional capability for finding the best solutions thanks to these special skills. Initially, a swarm with $h_{n}$ size of hummingbirds, as indicated in (5), is randomly assigned to $h_{n}$ food sources:

5 $$H_{i} = Lb + R \cdot \left( {Ub - Lb} \right),\quad i \in h_{n}$$

where ${\text{H}}_{{\text{i}}}$ refers to the location of the ith food source which depicts the PV cells parameters as a solution vector. The upper and lower bounds with problem dimension are demonstrated by ${\text{Ub}}$ and ${\text{Lb}}$; and R describes a randomized vector between [0, 1].

A visitation table (VT) of the food sources is established using the following criteria:

6 $$VT_{i,k} = \left\{ {\begin{array}{*{20}l} {null} \hfill & {if\, i = k} \hfill \\ 0 \hfill & {else} \hfill \\ \end{array} } \right., \forall i \in h_{n} ,\;and \;k \in h_{n}$$

where $VT_{i,k}$ shows how many times the ith hummingbird failed to reach the kth source of food, and null denotes the absence of any value.

Three flight maneuvers—axial, diagonal, and omnidirectional flights—are extensively used throughout foraging and modelled in the AHT which can each be seen in (7) as follows:

7 $$F^{\left( i \right)} = \left\{ {\begin{array}{*{20}l} {if\; r < \frac{1}{3}} \hfill & {\left\{ {\begin{array}{*{20}l} 1 \hfill & {if\; i = rand_{i} \left( {1,d} \right)} \hfill \\ 0 \hfill & {else} \hfill \\ \end{array} } \right.} \hfill & {} \hfill \\ {if\; \frac{1}{3} < r < \frac{2}{3}} \hfill & {\left\{ {\begin{array}{*{20}l} 1 \hfill & {if\; i = P\left( j \right),j \in \left[ {1,k\left] {,P = randperm\left( k \right),k \in } \right[2,} \right[r_{1} .\left( {d - 2} \right)\left] { + 1} \right]} \hfill \\ 0 \hfill & {else} \hfill \\ \end{array} } \right.} \hfill & {} \hfill \\ {Else} \hfill & {DF^{\left( i \right)} = 1} \hfill & {} \hfill \\ \end{array} } \right.\forall i \in d$$

where r1 denotes a randomly generated value falling inside [0, 1]; $rand_{i}$ and $randperm$ denotes randomized generating functions to create values in the form of integers and permuted integers, correspondingly.

The directed and territorial tactics of the hummingbirds randomly choose one of the flight skills described in (7). At first, a hummingbird uses the directed foraging method to inspect its intended source of food, which results in the discovery of a potential feed ingredient which is explained by:

8 $$Hnew_{i} \left( {t + 1} \right) = H_{i} \left( t \right) + a.DF.\left( {H_{i} \left( t \right) - H_{i,target} \left( t \right)} \right)$$

9 $${\varvec{a}} \sim {\varvec{N}}\left( {0,1} \right)$$

where $H_{i} \left( t \right)$ and $H_{i,t\arg et} \left( t \right)$ represent the positions of the present and intended i^th^ food sources at time t; N(0, 1) is the Gaussian distribution function.

Secondly, based on a certain territorial parameter (b), the territorial foraging strategy involves searching for a fresher food supply inside the surrounding area, as shown below:

10 $$Hnew_{i} \left( {t + 1} \right) = H_{i} \left( t \right) + b.DF.H_{i} \left( t \right)$$

11 $$b \sim N\left( {0,1} \right)$$

Therefore, the location of every food source (i) can be generally updated by:

12 $$H_{i} \left( {t + 1} \right) = \left\{ {\begin{array}{*{20}l} {Hnew_{i} \left( {t + 1} \right)} \hfill & {if \;O\left( {Hnew_{i} \left( {t + 1} \right)} \right) < O\left( {H_{i} \left( t \right)} \right)} \hfill \\ {H_{i} \left( t \right)} \hfill & {else} \hfill \\ \end{array} } \right. \quad \forall i \in h_{n}$$

where O (·) expresses the objective target described in (4). According to this model.

Third is the migratory foraging technique, in which hummingbirds would frequently fly into a more distant location to get foods whenever their region is food-scarce^41^. The hummingbirds could travel to different feeding sources, selected at random from the whole seeking area.

13 $$H_{worst} = Lb + r.\left( {Ub - Lb} \right)$$

where $H_{worst}$ denotes the source that has the minimum nectar replenishment rate within the population.

An inspection process should be performed ensuring that each hummingbird is always travelling inside the boundary searching space, and consequently each dimension variable that was abused, according to (14), will be returned to the search space boundary:

14 $$H_{i}^{\left( d \right)} \left( {t + 1} \right) = \left\{ {\begin{array}{*{20}l} {Lb^{\left( d \right)} ,} \hfill & { if \;H_{i}^{\left( d \right)} \left( {t + 1} \right) < Lb^{\left( d \right)} } \hfill \\ {Ub^{\left( d \right)} ,} \hfill & { if \;H_{i}^{\left( d \right)} \left( {t + 1} \right) > Ub^{\left( d \right)} } \hfill \\ {} \hfill H_{i}^{\left( d \right)} \left( {t + 1} \right),& else \hfill \\ \end{array} } \right.\quad \forall i \in h_{n} ,d \in \dim$$

The most pivotal element of the AHT is the visiting table which stores experience regarding food source visits. Accordingly, the visiting table can be updated for each hummingbird as depicted in (6).

14 $$VT_{i,k} = VT_{i,k} + 1,\quad if\; k \ne i\;\& \;k \ne target,\quad k \in h_{n}$$

15 $$VT_{i,target} = 0$$

16 $$VT_{i,k} = \mathop {\max }\limits_{{L \ne i \;\& \; L \in h_{n} }} \left( {VT_{i,L} } \right) + 1,\quad if\; k \ne i,\;k \in h_{n}$$

**Figure 3:**
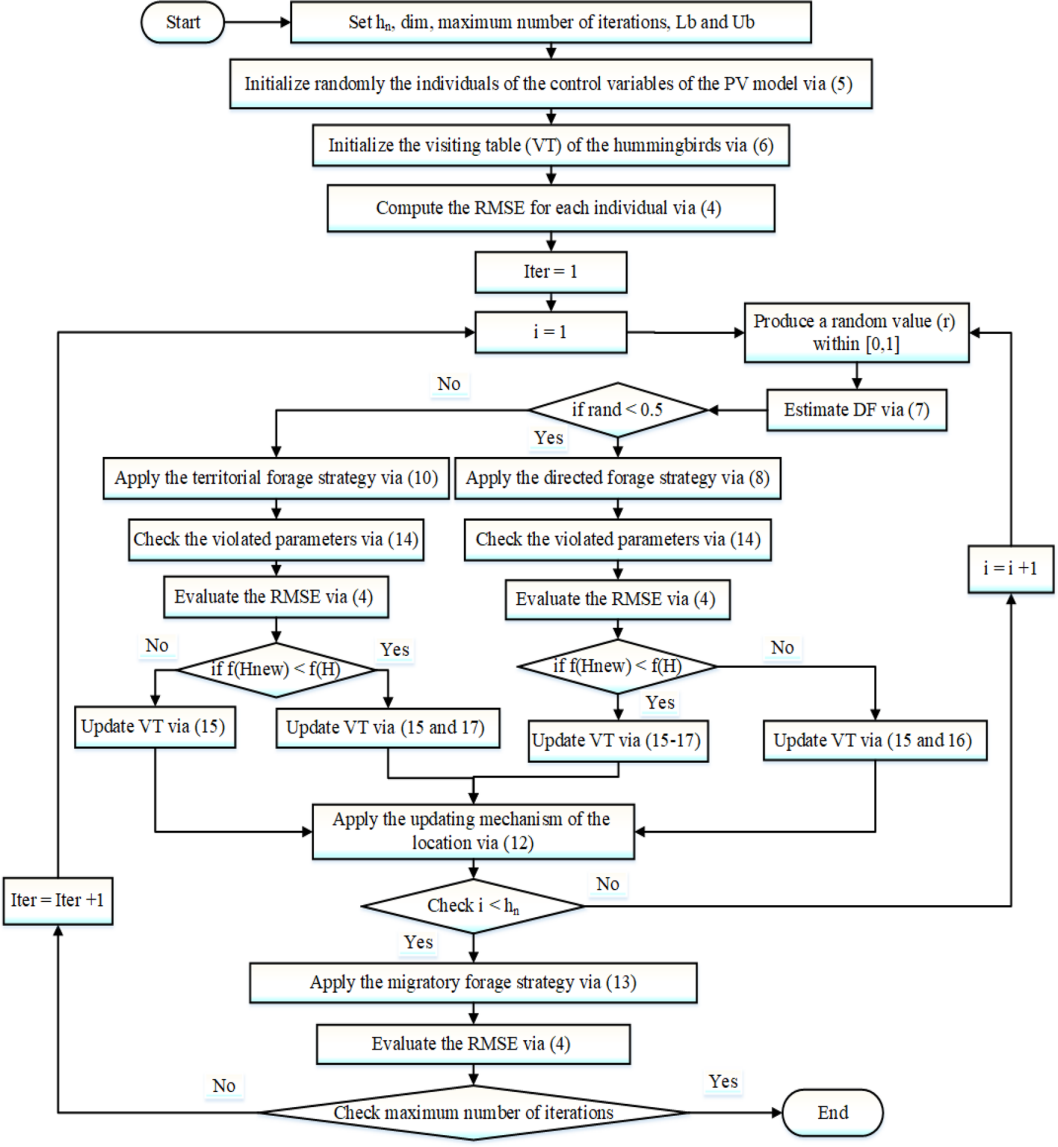
Main steps of the AHT for recognizing the unidentified parameters of PV cells.

The importance of this visiting table is to keep track of the time each food supply has passed without being revisited by a single bird, with a long gap between visits indicating a higher visit rate. Thus, Fig. 3 exhibits the principal steps of the AHT in recognizing the unidentified parameters of PV cells.

## Simulation results

The suggested AHT is carried out to identify the electrical SDM and DDM parameters for KC200GT and STM6-40/36. Two solar modules, which are multi-crystalline KC200GT with 54 cells in series and mono-crystalline STM6-40/36 with 36 cells^42^ in series at temperature of 25^◦^C and 51^◦^C, correspondingly, and an irradiance of (1000 W/m^2^). The measured data for KC200GT and STM6-40/36 contains 15 and 20 pairs of V/I values, respectively. Moreover, as depicted in Table 1, the lower (LB) and the upper (UB) bounds of parameters are demonstrated as per cell data.

**Table 1:** Bounded ranges per cell of the PV parameters.

Parameter	STM6-40/36		KC200GT		PWP 201	
	LB	UB	LB	UB	LB	UB
$I_{ph}$ (A)	0	2	0	9	0	1.5
$R_{S}$ (Ω)	0	0.36	0	0.5	0	0.5
$I_{SD1}$, $I_{SD2}$ (μA)	0	50	0	1	0	10
$n_{1}$, $n_{2}$	1	2	1	2	1	2
$R_{sh}$ (Ω)	0	1000	0	100	0	100

To compare the suggested AHT with some other newly established methods when used on the SDM and DDM of STM 6–40/36, four effectiveness metrics of maximum, average, minimum and standard deviation of the RMSE are displayed. Additionally, a low RMSE score shows that parameters were obtained effectively since RMSE aims to minimize the difference between measured and simulated data. Additionally, the AHT is validated on the PWP 201 polycrystalline PV module utilizing both SDM and DDM. The simulations are carried out with MATLAB 2017b on Intel® Core™ i7-7500U CPU @ 2.70 GHz 2.90 GHz with 8.00 GB RAM. Two scenarios are considered based on the selected objective function as follows:

- Scenario 1: Traditional objective model presented in Eq. (4a) for minimizing the error aggregation.
- Scenario 2: minimization of the (MAE) objective function presented in Eq. (4b).

### STM6-40/36 PV module

#### Simulated results of scenario 1 for STM6-40/36 PV module

The specific parameters of SDM and DDM of STM6-40/36 are estimated by the proposed AHT and AVO^33^, TSO^32^, and TLSBO^43^ that are implemented in this article for the first time as depicted in Table 2. In terms of the numerical simulations, for SDM, the proposed AHT could achieve the lowest possible value of 1.7298E−3, whilst AVO, TSO, and TLSBO achieve the lowest possible values of 1.7324E−3, 1.9219E−3, and 1.9264E−3, respectively as manifested in Table 2. The AHT could achieve the lowest possible value of 1.7028E−3, whilst AVO, and TSO achieve the lowest possible values of 1.7049E−3 and 2.6843E−3, accordingly for DDM.

**Table 2:** Extracted parameters by the compared algorithms for STM6-40/36 PV module of Scenario 1 (results are reported per cell).

Parameter	SDM				DDM		
	AVO	TLSBO	TSO	AHT	AVO	TSO	AHT
$I_{ph}$ (A)	1.663771676	1.662558762	1.662005036	1.66390477	1.663536287	1.663208295	1.663752058
$R_{S}$ (Ω)	0.004149226	0.003385381	0.003674857	0.004273775	0.0054189	0.003700979	0.005187934
$R_{S}$ $R_{Sh}$ (Ω)	16.16351325	18.71897053	18.86505402	15.92830436	17.40956979	20.23228291	16.82021479
$I_{SD1}$ (μA)	1.80959E−6	2.36288E−6	2.19825E−6	1.73866E−6	7.57976E−6	2.07517E−8	6.00651E−7
$n_{1}$	1.524708873	1.554677592	1.546432872	1.520302863	1.980144605	1.217207105	1.424988503
$I_{SD2}$ (μA)	–	–	–	–	4.11054E−7	6.86515E−6	6.26664E−6
$n_{2}$	–	–	–	–	1.393882913	1.752751642	1.998145081
RMSE	1.7324E−3	1.9264E−3	1.9219E−3	1.7298E−3	1.7049E−3	2.6843E−3	1.7028E−3

**Figure 4:**
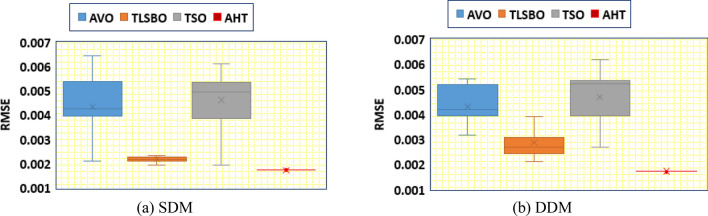
Whisker’s plot of the AHT in comparison with AVO, TLSBO, and TSO with the two models of STM6-40/36 (Scenario 1).

In addition, thirty independent runs are conducted for the proposed AHT, AVO, TSO, and TLSBO for SDM and DDM, in this article, to show the performance of these optimizers. It can be noticed from conducting these runs that the proposed AHT has the minimum value among these techniques which highlight the efficiency and robustness of the proposed AHT compared with these optimizers as exemplified in Fig. 4. As shown, for SDM, AHT derives the least minimum, mean, maximum and standard deviations related to the RMSE of 0.001729814, 0.001729831, 0.001730045 and 5.39233E−8, respectively. On contrary, AVO, TLSBO and TSO obtain higher standard deviations of 0.001084, 0.000102 and 0.000967, respectively. Similar findings are acquired for DDM, AHT achieves the least minimum, mean, maximum and standard deviations related to the RMSE of 0.001704932, 0.001728661, 0.001762892 and 9.85118E−6, respectively. On contrary, AVO, TLSBO and TSO, respectively, obtain higher standard deviations of 0.000833907, 0.000537932 and 0.000899015.

**Figure 5:**
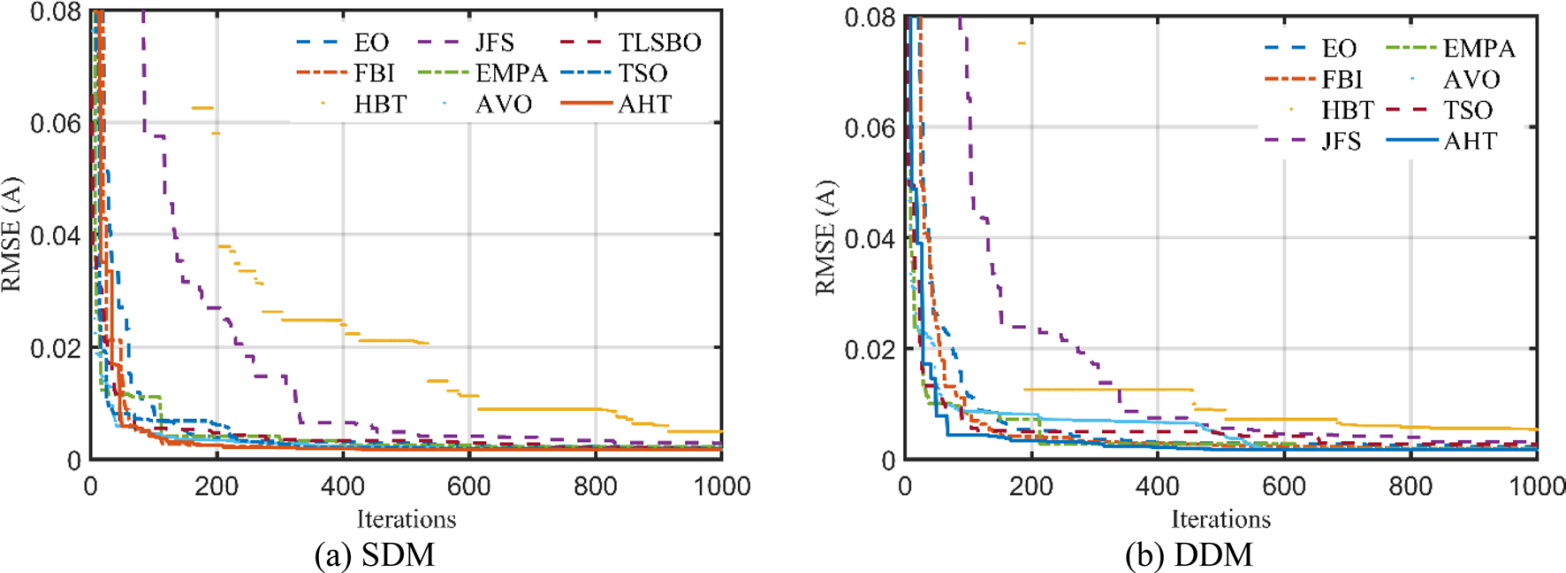
Convergence characteristics of the compared algorithms for the two models of STM6-40/36 (Scenario 1).

The convergence characteristics of AHT are developed for SDM and DDM as illustrated in Fig. 5a,b and compared to AVO, TSO, TLSBO, Forensic-Based Investigation (FBI) Technique^44^, Enhanced marine predator approach (EMPA)^45^, Equilibrium Optimization (EO), Heap-based Technique^29,46,47^, and Jellyfish search (JFS) optimizer^48^. It can be manifested from this figure that the convergence characteristics of the AHT has an excellent performance contrasting to these optimizers.

**Figure 6:**
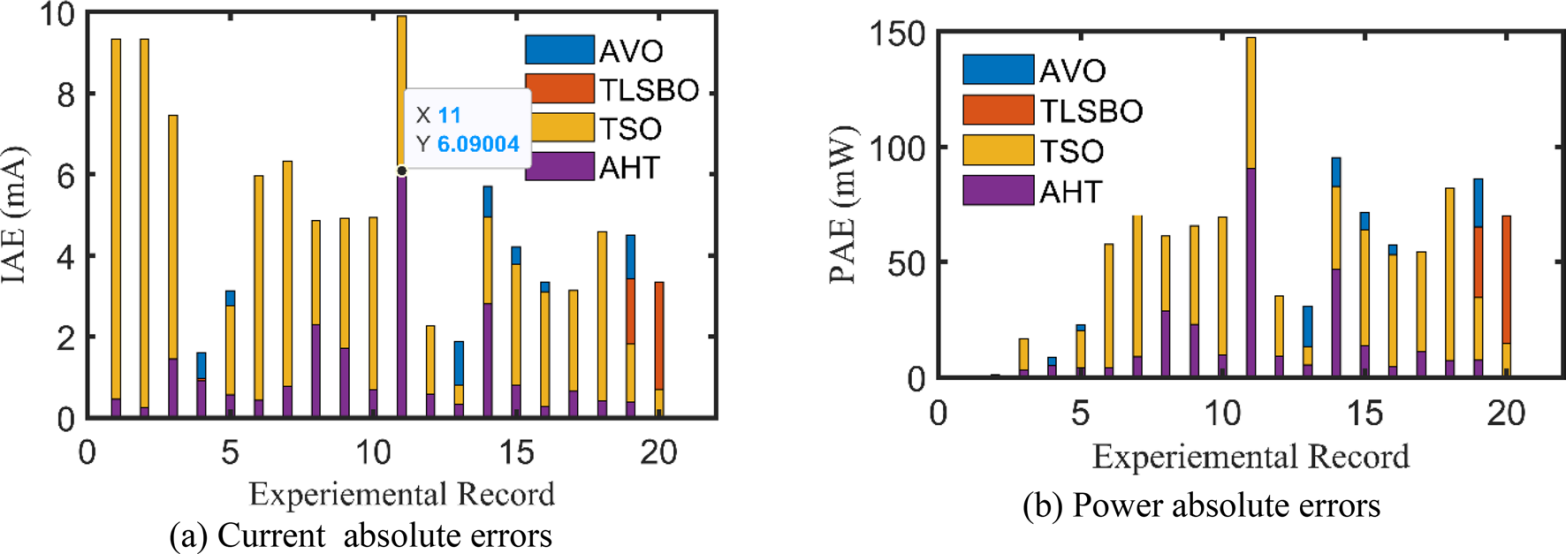
Absolute errors cropped by the AHT and other optimizers for SDM of STM6-40/36 PV module (Scenario 1).

**Figure 7:**
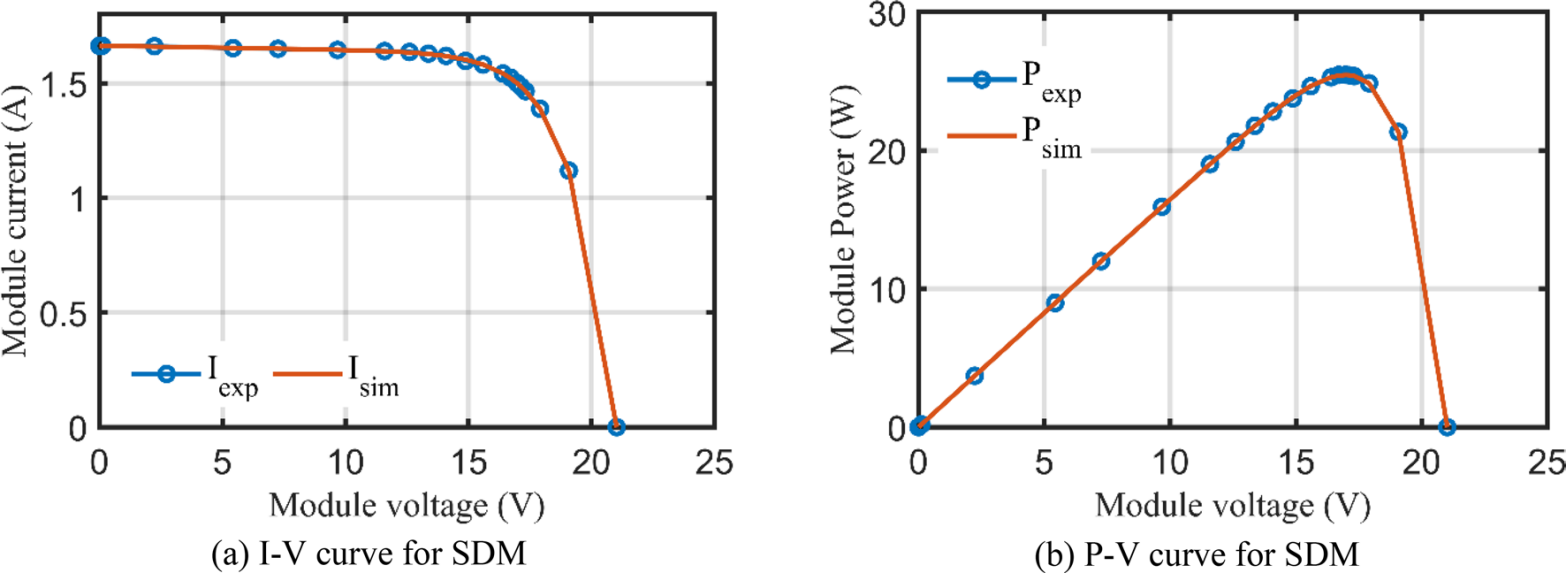
The I–V and P–V curves developed by the proposed AHT for SDM of STM6-40/36 PV module (Scenario 1).

For SDM, the experimental and estimated data illustrated by AHT, AVO, TSO, and TLSBO of the currents and powers are pronounced in Fig. 6a,b, respectively for each 20 points. Besides, at each point, the absolute error between estimated and experimental data for the currents and powers are exemplified as manifested in the previously mentioned figures, where the proposed AHT achieves the lowest absolute errors in comparison with AVO, TSO, and TLSBO. For sake of quantifications, as demonstrated in Fig. 6a, the maximum absolute percentage errors between measured and estimated current values is 0.14% at the experimental point no. 11 with IAE value of less than 6.09 mA cropped by the AHT. The I–V curve and the P–V curve are illustrated in Fig. 7a,b, respectively which exemplifies the precise proximity between both the estimated and experimental data of the powers and currents at each point of voltage.

**Figure 8:**
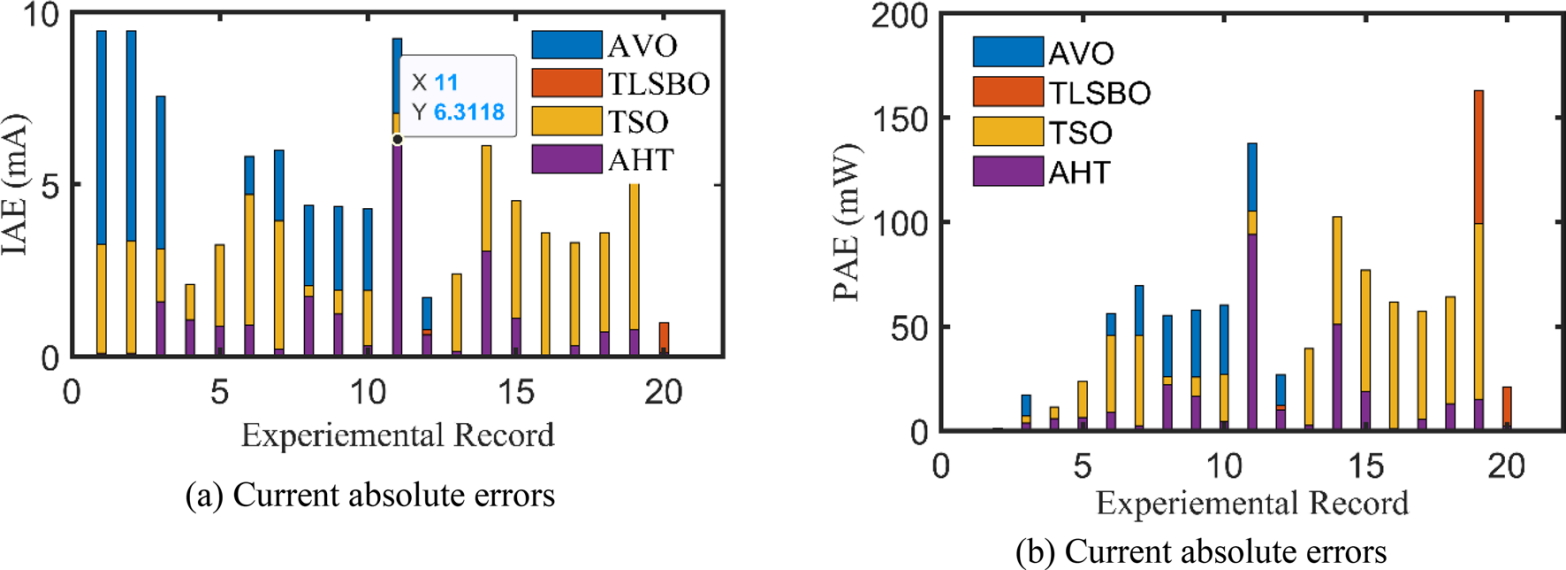
Absolute errors produced by the AHT and other optimizers for DDM of STM6-40/36 PV module (Scenario 1).

**Figure 9:**
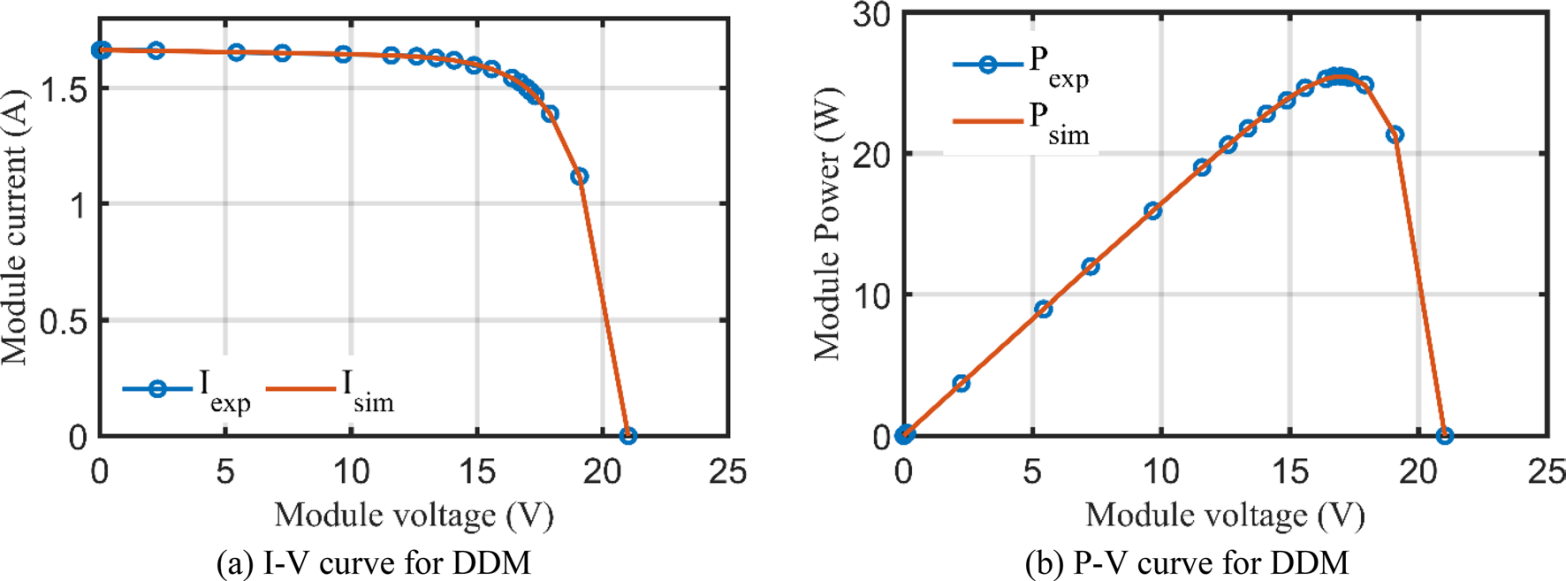
The I–V and P–V curves developed by the AHT for DDM of STM6/40-36 PV module (Scenario 1).

For DDM, the experimental data and the estimated data illustrated by AHT, AVO, TSO, and TLSBO of the currents and powers are described in Fig. 8(a)-(b), respectively for each 20 points. Besides, at each point, the absolute error between estimated and experimental data for the currents and powers are exemplified as manifested, where the proposed AHT achieves the lowest absolute errors in comparison with AVO, TSO, and TLSBO. Once again, as indicated in Fig. 8(a), the maximum absolute percentage errors between measured and estimated current values is 0.40% with IAE value of less than 6.31 mA cropped by the AHT. The I–V curve and the P–V curve are illustrated in Fig. 9a,b which exemplifies the closeness between both the estimated and experimental data of the powers and currents at each point of voltage.

The statistical analysis including Best, Worst, Average, and Standard deviation parameters for SDM and DDM are demonstrated in Tables 3 and 4, correspondingly. In these tables, the proposed AHT is compared to several optimizers of AVO, TSO and TLSBO, and the reported optimizers of simulated annealing (SA)^49^, three point based approach (TPBA)^50^, hybridizing cuckoo search/biogeography based optimization (BHCS)^51^, ITLBO^25^, enhanced CS approach (ECSA)^52^, improved shuffled complex evolution (ISCE)^53^, chaotic logistic Rao technique (CLRT)^54^, enhanced PSO (EPSO)^55^, fractional chaotic PSO (FC-EPSO)^56^, bat optimization approach (BA), novel BA (NBA), directional bat algorithm (DBA)^57^, SDO^58^, MPA^59^ and improved chaotic whale optimization algorithm (ICWOA)^61^ for both models.

**Table 3:** Comparative assessment of the compared optimizers for SDM of STM6-40/36 PV module (Scenario 1).

Optimizer	Min (RMSE)	Mean (RMSE)	Max (RMSE)	Std (RMSE)	Population size	Maximum number of iterations
AHT	1.7298E−3	1.7298E−3	1.7300E−3	5.3923E−8	100	1000
AVO	1.7324E−3	4.3485E−3	6.4338E−3	1.0842E−3	100	1000
TLBSO	1.9264E−3	2.1765E−3	2.3319E−3	1.0236E−4	100	1000
TSO	1.9219E−3	4.6229E−3	6.1168E−3	9.6669E−4	100	1000
ISCE^53^	1.7300E−3	1.7298E−3	1.7298E−3	–	22	–
TPBA^50^	1.7740E−3	–	–	–	50	50
ITLBO^25^	1.7300E−3	1.7300E−3	1.7300E−3	–	50	1000
SA^49^	3.3990E−3	–	–	–	30	250
Hunter algorithm^60^	1.7298E−3	–	–	–	200	1000
BHCS^51^	1.7298E−3	1.8365E−3	3.3299E−3	–	10	250
ECSA^52^	1.7946E−3	1.7943E−3	1.7943E−3	–	25	1500
SDO^58^	1.7300E−3	1.7300E−3	1.7300E−3	4.3700E−18	100	1000
MPA^59^	1.781E−3	–	–	–	–	5000
ICWOA^61^	1.9E−3	–	–	–	150	10,000

**Table 4:** Comparative assessment of the compared optimizers for DDM for STM6-40/36 PV module (Scenario 1).

Optimizer	Min (RMSE)	Mean (RMSE)	Max (RMSE)	Std (RMSE)	Population number	maximum number of iterations
AHT	1.7049E−3	1.7287E−3	1.7629E−3	9.8512E−6	100	1000
AVO	1.7028E−3	4.3163E−3	5.4137E−3	8.3391E−4	100	1000
TSO	2.6843E−3	4.6888E−3	6.1670E−3	8.9902E−4	100	1000
EPSO^55^	1.8307E−3	–	–	–	1000	100
CLRT^54^	1.7120E−3	–	–	–	10	1000
FC-EPSO^56^	1.7720E−3	–	–	–	30	200
DBA^57^	1.7320E−3	4.9340E−3	1.3728E−2	2.8930E−3	40	5000
BA^57^	2.1946E−2	9.2023E−2	1.4481E−2	2.4070E−2	50	5000
NBA^57^	1.8268E−3	4.1404E−3	7.5980E−3	1.4300E−3	50	5000
SDO^58^	1.7252E−3	1.8453E−3	2.9616E−3	1.9000E−4	100	1000
MPA^59^	1.7780E−3	–	–	–	–	5000

As demonstrated, the AHT achieves the least RMSE, standard deviation, mean and maximum of 1.7298E−3, 5.3923E−8, 1.7298E−3, 1.7300E−3, respectively, for SDM (see Table 3). The AHT achieves 1.7049E−3, 9.8512E−6, 1.7287E−3, and 1.7629E−3, respectively for DDM as indicated in Table 4. The comparative assessment exemplifies high search accuracy and good stability of the suggested AHT compared to several newly techniques and the reported optimizers.

#### Simulated results of scenario 2 for STM6-40/36 PV module

**Figure 10:**
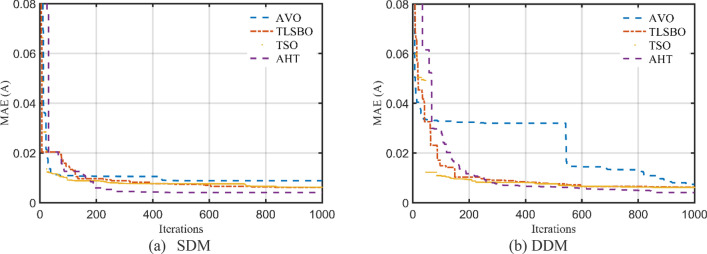
Convergence characteristics of the compared algorithms for the two models of STM6-40/36 (Scenario 2).

For this scenario, the proposed AHT, AVO, TSO, and TLSBO are implemented and the regarding parameters of SDM and DDM of STM6-40/36 are depicted in Table 5. Also, the convergence characteristics of the AHT in comparison with AVO, TLSBO, and TSO for this scenario are developed for SDM and DDM as illustrated in Fig. 10a,b. In terms of the numerical simulations, for SDM, the proposed AHT could achieve the lowest MAE value of 4.068E−3, whilst AVO, TSO, and TLSBO achieve the lowest possible values of 8.805E−3, 6.175E−3 and 6.193E−3, respectively as manifested in Table 5. The AHT could achieve the lowest MAE value of 3.99E−3, whilst AVO, TSO, and TLSBO achieve the lowest possible values of 7.291E−3, 6.243E−3 and 6.108E−3, accordingly for DDM.

**Table 5:** Extracted parameters by the compared algorithms for STM6-40/36 PV module of Scenario 2 (results are reported per cell).

Parameter	SDM				DDM			
	AVO	TLSBO	TSO	AHT	AVO	TLSBO	TSO	AHT
$I_{ph}$ (A)	1.654201	1.657288	1.657691	1.665287	1.660206	1.657262	1.657037	1.663651
$R_{S}$ (Ω)	7.1800E−7	0.000529	0.0000	0.003682	1.0700E−8	0.000388	9.5600E−6	0.004382
$R_{Sh}$ (Ω)	672.4723	29.35333	34.6015	13.89569	46.02487	34.77217	35.85202	15.61299
$I_{SD1}$ (μA)	8.9700E−6	5.0100E−6	6.0800E−6	1.8200E−6	6.8800E−8	5.5800E−6	0.0000	6.6100E−7
$n_{1}$	1.724449	1.645837	1.671148	1.525607	1.352792	1.659659	1.852299	1.4356
$I_{SD2}$ (μA)	–	–	–	–	1.1500E−5	7.21E−10	5.9700E−6	5.9910E−6
$n_{2}$	–	–	–	–	1.797415	1.820005	1.668591	1.9756445
MAE	8.805E−3	6.175E−3	6.193E−3	4.068E−3	7.291E−3	6.243E−3	6.108E−3	3.990E−3

**Figure 11:**
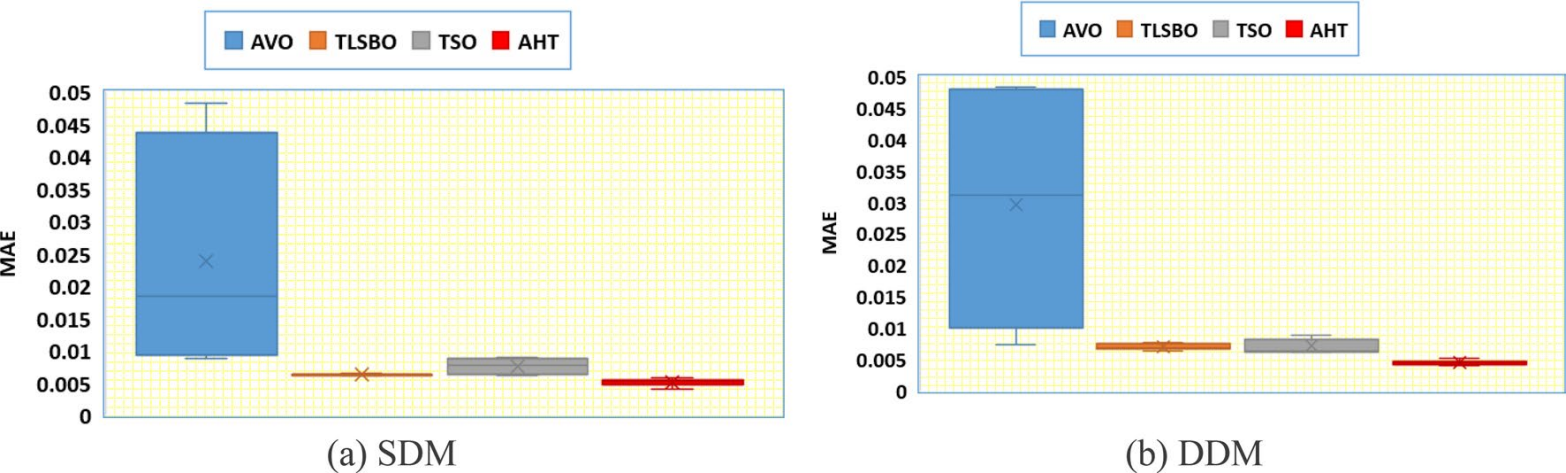
Whisker’s plot of the AHT in comparison with AVO, TLSBO, and TSO with the two models of STM6-40/36 (Scenario 2).

In addition, Fig. 11 displays the regarding Whisker’s plot of the AHT in comparison with AVO, TLSBO, and TSO for this scenario. As shown, for SDM, AHT derives the least mean value related to the MAE of 0.005084. On contrary, AVO, TLSBO and TSO obtain higher MAE objectives of 0.023869, 0.006279 and 0.007603, respectively. For DDM, AHT derives the least minimum, mean, maximum and standard deviation related to the MAE of 0.00399, 0.004383, 0.005025 and 0.000299, respectively. On contrary, AVO, TLSBO and TSO obtain higher standard deviations of 0.017904, 0.000501 and 0.001165, respectively.

**Figure 12:**
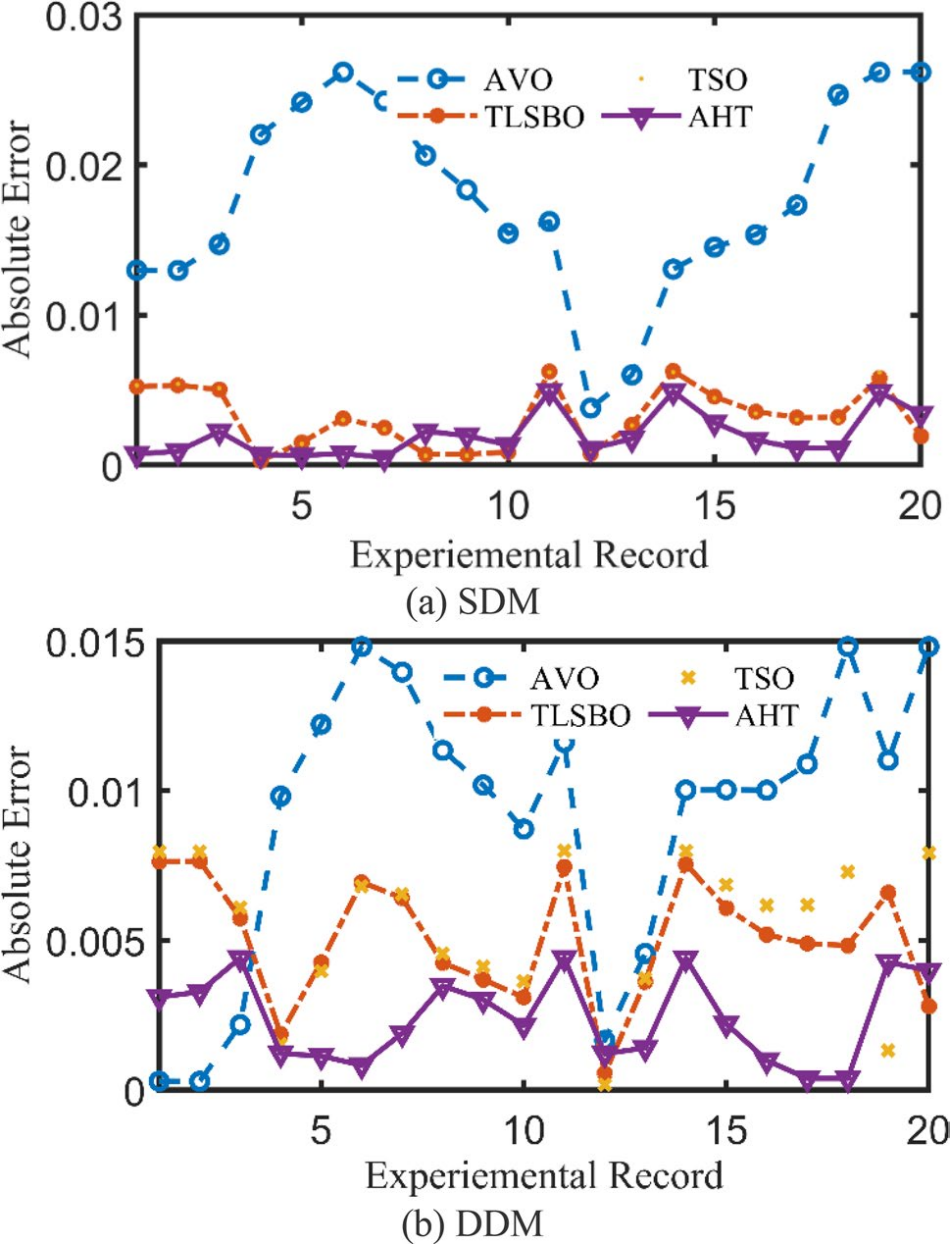
Absolute errors in the produced current by the compared optimizers of STM6-40/36 PV module (Scenario 2).

For both models at this scenario, the experimental absolute errors in the produced current are described in Fig. 12a,b for the AHT, AVO, TLSBO, and TSO, respectively for each 20 points. As shown, the proposed AHT derives superior capability compared to the others in minimizing the maximal absolute error. Based on this scenario, the distribution of errors is approximately equivalent and more suitable where the searching direction is dedicated for minimizing the maximum error over the course of the experimental recordings. For the SDM, the errors using the proposed AHT range from 0.000495 at the reading no. 7 to 0.004901 at the reading no. 11. Therefore, the regarding difference between the two obtained boundaries is 0.004406. In similar way, the calculated difference between the two obtained boundaries using AVO, TLSBO, and TSO are 0.02237, 0.005984 and 0.006012, respectively. These differences demonstrate the high capability of the proposed AHT in achieving the best distribution of the errors over the course of the experimental recordings.

For the DDM, the errors using the proposed AHT range from 0.000389 at the reading no. 18 to 0.004415 at the reading no. 11. The regarding difference between the two obtained boundaries is 0.00403. In similar way, the calculated difference between the two obtained boundaries using AVO, TLSBO, and TSO are 0.01452, 0.007083 and 0.007797, respectively. These differences demonstrate the high capability of the proposed AHT in achieving the best distribution of the errors over the course of the experimental recordings.

### KC200GT PV module

#### Simulated results of scenario 1 for KC200GT PV module

The specific parameters of SDM and DDM of KC200GT PV module are estimated by the proposed AHT and recently developed optimizers which are AVO^33^, TSO^32^, TLSBO^62^ that are implemented in this article for the first time as depicted in Table 6. Besides, for SDM, the proposed AHT could achieve the lowest possible value of 6.4957E−4, whilst AVO, TSO, and TLSBO achieve the lowest possible values of 1.0426E−2, 1.1538E−2, and 1.2897E−2, respectively. It is also seen from this table that the proposed AHT could achieve the lowest possible value of 3.7154E−4, whilst AVO, TSO, and TLSBO achieve the lowest possible values of 9.6100E−3, 1.1268E−2 and 1.2580E−2, respectively for DDM.

**Table 6:** Extracted parameters by the compared algorithms for KC200GT PV module of Scenario 1 (Reported values are per cell).

Parameters	SDM				DDM			
	AVO	TLSBO	TSO	AHT	AVO	TLSBO	TSO	AHT
$I_{ph}$ (A)	8.196860511	8.195632092	8.184234558	8.216563341	8.194960209	8.227019407	8.187041565	8.215974788
$R_{S}$ (Ω)	0.00461085	0.004621431	0.004729641	0.004823304	0.004739349	0.004615996	0.004818806	0.004847758
$R_{Sh}$ (Ω)	15.82435839	24.17854623	31.18677215	6.319972337	19.13139357	6.763110605	40.57587312	6.520483032
$I_{SD1}$ (μA)	5.93825E−8	6.14808E−8	4.2582E−8	2.64674E−8	1.15508E−8	8.04852E−8	9.63558E−7	6.14823E−7
$n_{1}$	1.265408578	1.267770444	1.243359198	1.213470219	1.173682701	1.818799115	1.788554316	1.931107246
$I_{SD2}$ (μA)	–	–	–	–	7.80931E−7	4.68331E−8	1.81127E−8	2.08875E−8
$n_{2}$	–	–	–	–	1.655378412	1.249918157	1.194190552	1.199926243
RMSE	1.0426E−2	1.2897E−2	1.1538E−2	6.4957E−4	9.6100E−3	1.2580E−2	1.1268E−2	3.7154E−4

**Figure 13:**
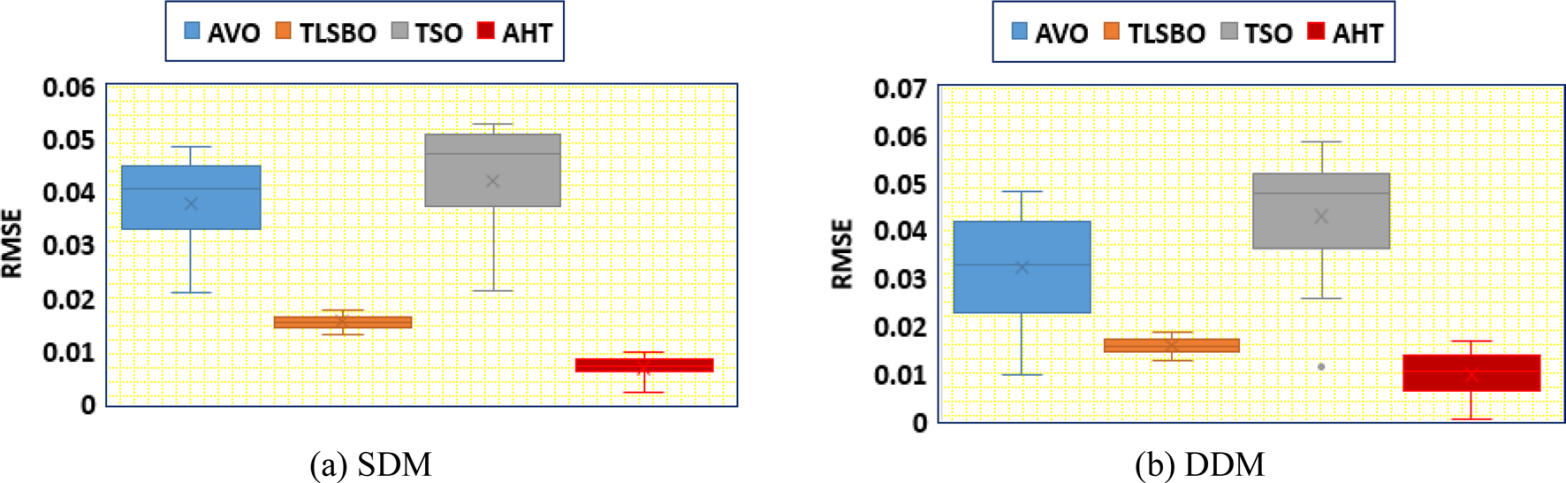
Whisker’s plot of the AHT in comparison with AVO, TLSBO, and TSO with the two models of KC200GT PV module (Scenario 1).

Thirty independent runs are conducted for the proposed AHT, AVO, TSO, and TLSBO for SDM and DDM of this module, in this article, to show the performance of these optimizers. It can be noticed from conducting these runs that the proposed AHT has the minimum value among these techniques which highlight the efficiency and robustness of the proposed AHT compared with these optimizers as exemplified in Fig. 13.

As shown, for SDM, AHT acquires the least minimum, mean, maximum and standard deviations related to the RMSE of 0.0006496, 0.0067283, 0.0095589 and 0.0023414, respectively. On contrary, AVO, TLSBO and TSO obtain higher mean RMSE of 0.037613, 0.015379 and 0.041981, respectively. As well, they obtain higher standard deviations of 0.009989, 0.00121 and 0.011769, respectively. Similar findings are acquired for DDM, AHT obtains the least minimum, mean, maximum and standard deviations related to the RMSE of 0.000371545, 0.009679481, 0.016820415 and 0.005198108, respectively. On contrary, AVO, TLSBO and TSO, respectively, obtain higher standard deviations of 0.011457421, 0.001713595 and 0.013369672.

**Figure 14:**
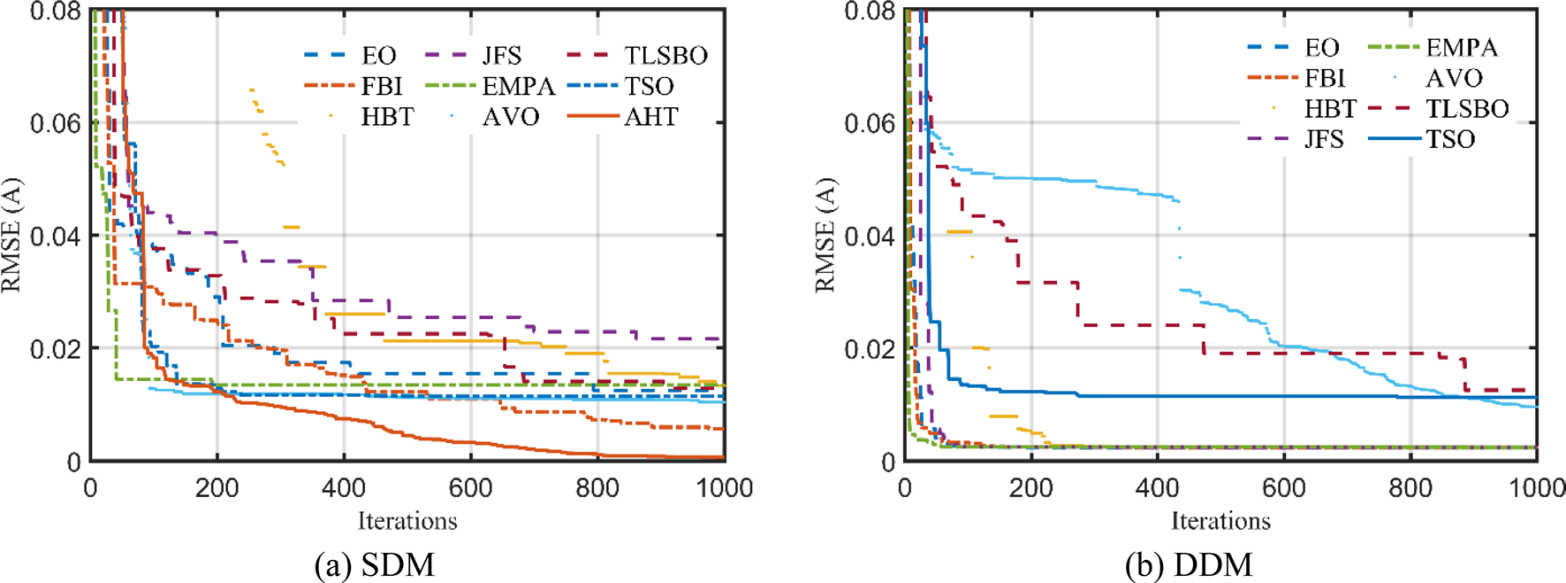
Convergence characteristics of the AHT in comparison with other optimizers of KC200GT PV module (Scenario 1).

The convergence characteristics of AHT for SDM and DDM are illustrated in Fig. 14a,b, respectively and compared to AVO, TSO, TLSBO, EMPA, EO, Heap, JFS and FBI^44^. It can be manifested from this figure that the convergence characteristics of the AHT has an excellent performance in comparison with these optimizers.

**Figure 15:**
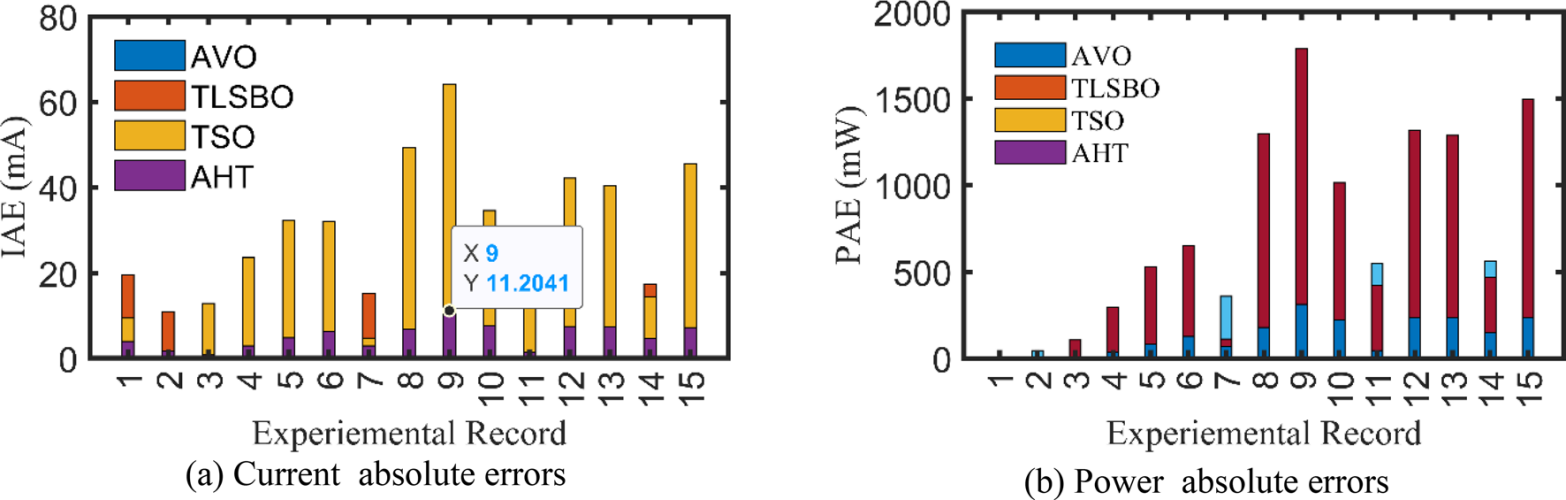
Absolute errors cropped by the AHT and other optimizers for SDM of KC200GT PV module (Scenario 1).

**Figure 16:**
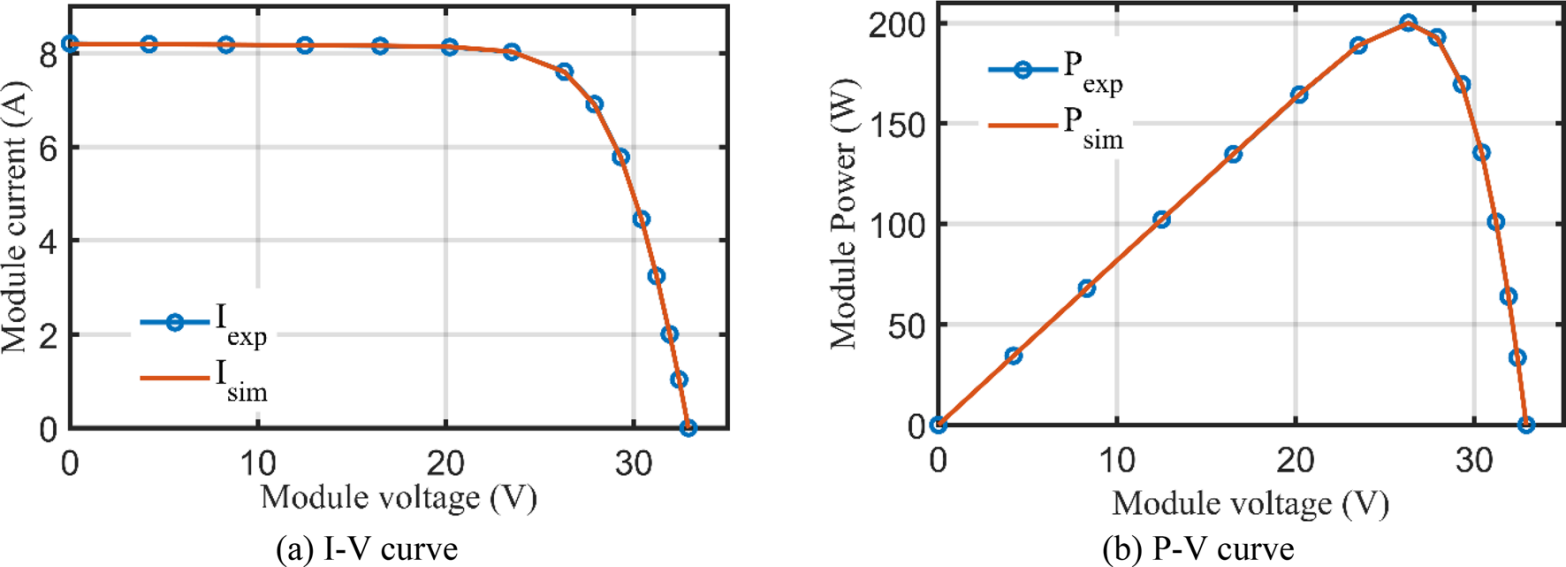
The I–V and P–V curves developed by the AHT for SDM of KC200GT PV module (Scenario 1).

For SDM, the experimental data and the estimated data illustrated by AHT, AVO, TSO, and TLSBO of the currents and powers are described in Fig. 15a,b, respectively for each 15 points. Besides, at each point, the absolute error between estimated and experimental data for the currents and powers are exemplified as manifested, where the proposed AHT achieves the lowest absolute errors in comparison with AVO, TSO, and TLSBO. For sake of quantifications, as shown in Fig. 15, the maximum absolute percentage errors between measured and estimated current values is 0.45% at the experimental point no. 9 with IAE value of 11.2 mA cropped by AHT. The I–V curve and the P–V curve are illustrated in Fig. 16a,b which exemplifies the closeness between both the estimated and experimental data of the powers and currents at each point of voltage.

**Figure 17:**
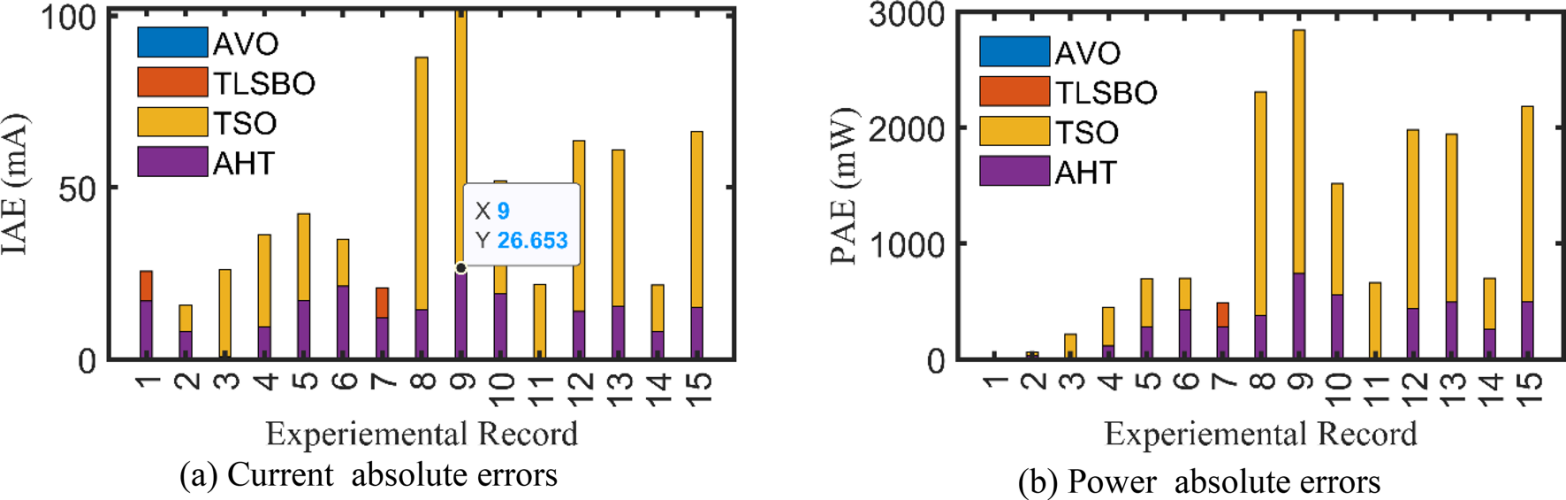
Absolute errors proposed by the AHT and other optimizers for DDM of KC200GT PV module (Scenario 1).

**Figure 18:**
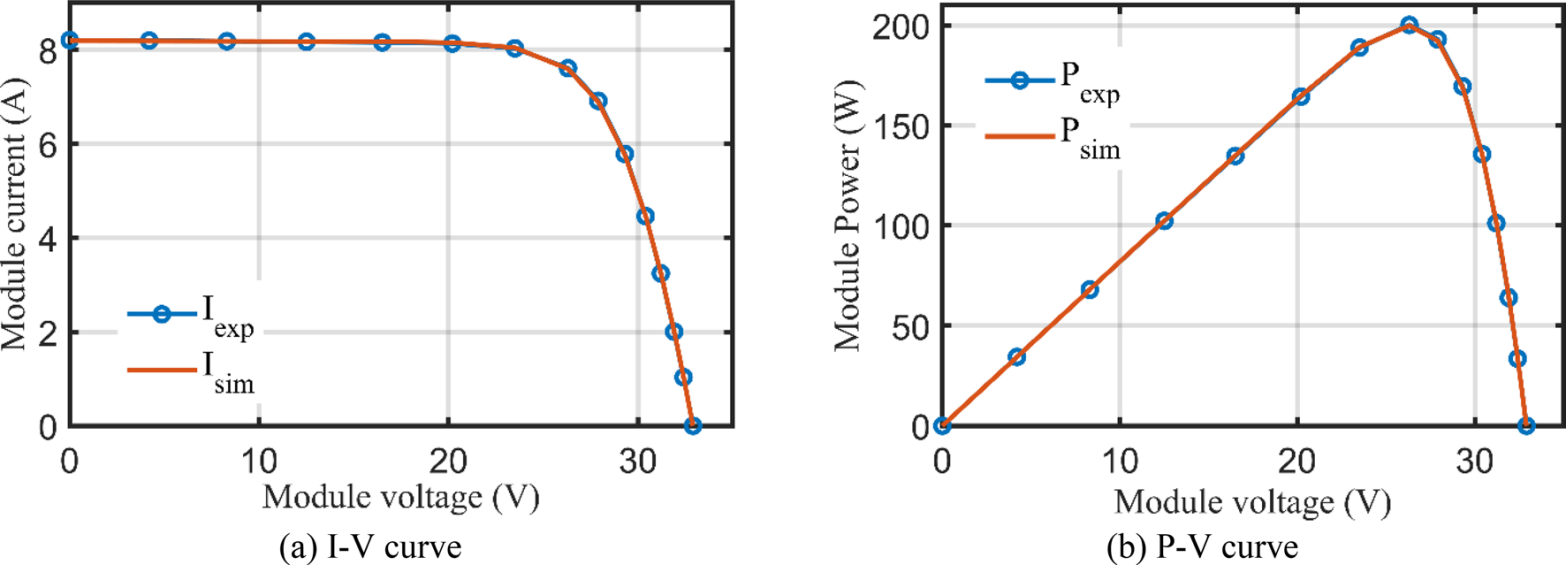
The I–V curve and P–V curve developed by the AHT for DDM of KC200GT PV module (Scenario 1).

For SDM, the experimental data and the estimated data illustrated by AHT, AVO, TSO, and TLSBO of the currents and powers are described in Fig. 17a,b, respectively for each 15 points. Besides, at each point, the absolute error between estimated and experimental data for the currents and powers are exemplified, where the proposed AHT achieves the lowest absolute errors in comparison with AVO, TSO, and TLSBO. The I–V curve and the P–V curve are illustrated in Fig. 18a,b which exemplifies the closeness between both the estimated and experimental data of the powers and currents at each point of voltage.

For the KC200GT PV module, the statistical analysis for SDM and DDM are demonstrated in Tables 7 and 8, respectively. In this Table, the proposed AHT is compared to AVO, TSO and TLSBO, and the reported optimizers which are CPMPSO^63^, PSO^64^, BMA^65^, NLBMA^66^, PGJAYA^67^, FPSO^68^, Hybridized Pattern Search and Firefly Technique (HPSFT)^69^, FBI^44^, EHHO^70^, and MVO^71^ for both models. As shown, the suggested AHT achieves the smallest RMSE, standard deviation, mean and maximum of 6.4957E−4, 2.3414E−3, 6.7283E−3, and 9.5589E−3, respectively, for SDM as depicted in Table 7. On the other hand, the AHT achieves 3.7154E−4, 5.1981E−3, 9.6795E−3, and 1.6820E−2, respectively for DDM (See Table 8). The comparative assessment exemplifies high search accuracy and good stability of the proposed AHT in comparison with the recently developed optimizers and the reported optimizers.

**Table 7:** Comparative assessment of the compared optimizers for KC200GT PV module using SDM (Scenario 1).

Optimizer	Max (RMSE)	Mean (RMSE)	Min (RMSE)	Std (RMSE)	Population number	Maximum number of iterations
AHT	9.5589E−3	6.7283E−3	6.4957E−4	2.3414E−3	100	1000
AVO	4.8434E−2	3.7613E−2	1.0426E−2	9.9886E−3	100	1000
TLSBO	1.7537E−2	1.5379E−2	1.2897E−2	1.2096E−3	100	1000
TSO	5.2761E−2	4.1981E−2	1.1538E−2	1.1769E−2	100	1000
FBI^44^	–	–	7.3400E−4	–	100	1000
CPMPSO^63^	–	–	1.53903E−3	–	30	5000
PSO^64^	5.3291E−1	3.4467E−1	1.0195E−1	–	–	500
NLBMA^66^	3.3610E−2	3.3610E−2	3.3610E−2	–	20	200
PGJAYA^67^	–	–	1.5455E−4	–	20	5000
BMA^65^	1.4986E−1	1.2442E−1	1.0244E−1	–	30	500
FPSO^68^	–	–	2.8214E−2	–	100	10,000
HPSFT^69^	–	–	4.9863E−2	–	50	5000
EHHO^70^	–	–	5.9507E−2	–	30	1000
MVO^71^	–	–	8.3800E−2	–	20	100
SNS^72^	1.4790E−2	1.2558E−2	8.8035E−3	1.4612E−3	100	1000

**Table 8:** Comparative assessment of the compared optimizers for KC200GT PV module using DDM (Scenario 1).

Optimizer	Max (RMSE)	Mean (RMSE)	Min (RMSE)	Std (RMSE)	Population number	Maximum number of iterations
AHT	1.6820E−2	9.6795E−3	3.7154E−4	5.1981E−3	100	1000
AVO	4.7919E−2	3.2127E−2	9.6100E−3	1.1457E−2	100	1000
TLSBO	1.8783E−2	1.5791E−2	1.2580E−2	1.7136E−3	100	1000
TSO	5.8583E−2	4.2900E−2	1.1268E−2	1.3370E−2	100	1000
PSO^64^	7.9194E–1	4.5668E−1	1.2970E−1	–	−	500
BMA^65^	3.0902E–1	2.1858E−1	1.2492E−1	–	30	500
NLBMA^66^	3.3043E–2	3.3043E−2	3.3043E−2	–	20	200
FBI^44^	–	–	9.6200E−4	–	100	1000
SNS^72^	2.1884E−2	1.5129E−2	8.5517E−3	2.4465E−3	100	1000

**Figure 19:**
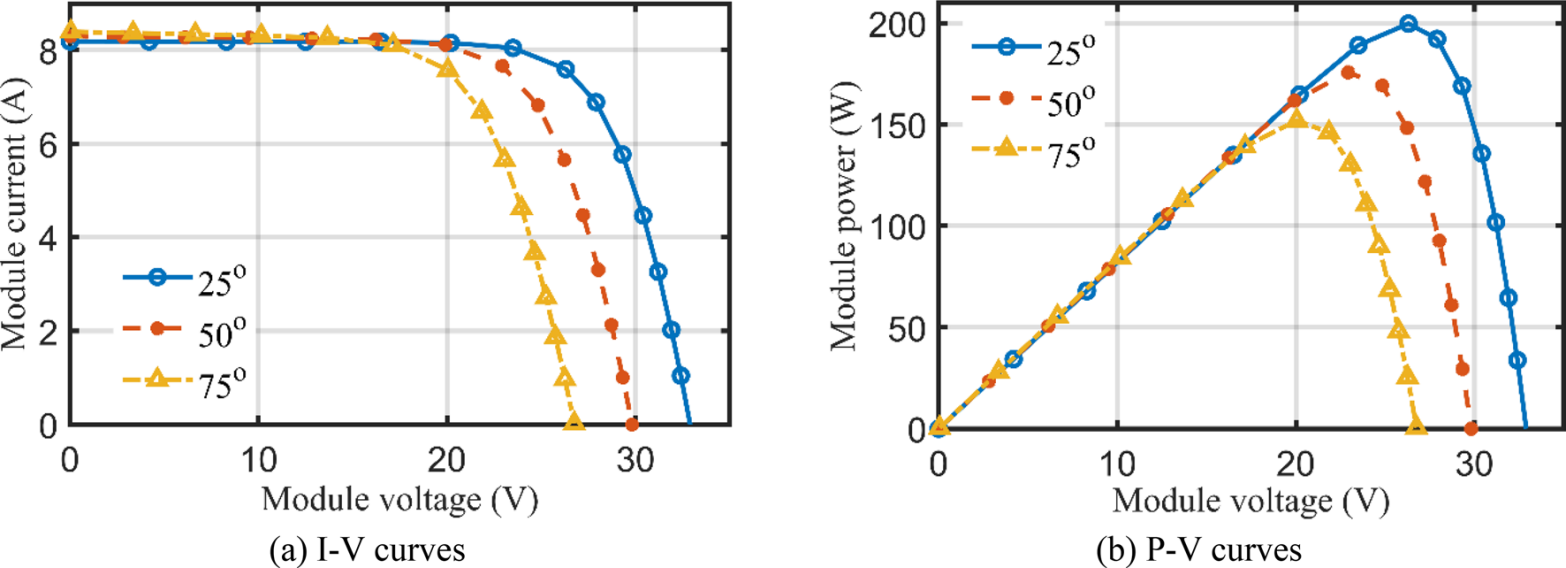
Principal characteristics of KC200GT PV module with temperature variations at 1000 W/m^2^ irradiance.

**Figure 20:**
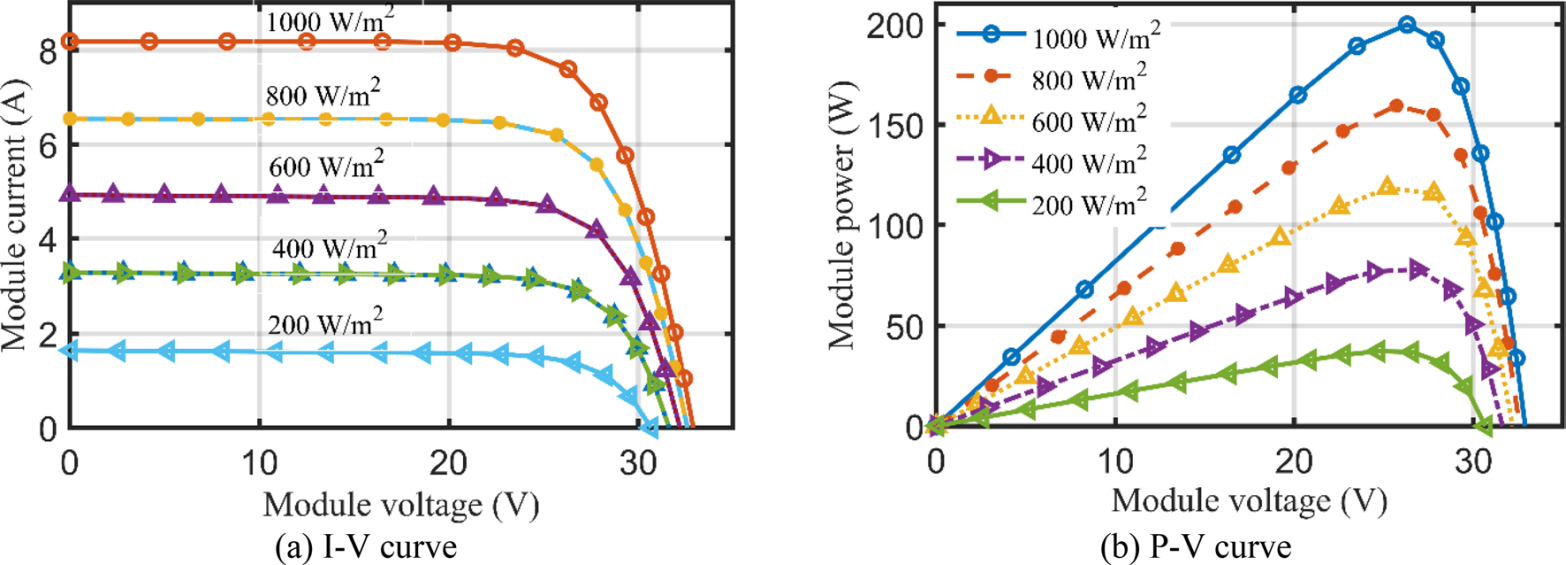
Principal characteristics of KC200GT PV module with irradiance variations at temperature of 25 °C.

To validate the AHT results at different temperatures and at different solar radiations, Figs. 19a,b and 17a,b provide the corresponding I–V and P–V curves for KC200GT PV module. With temperature variations at irradiance level of 1000 W/m^2^, as shown in Fig. 19a,b, the proposed AHT derives significant coincidence between the simulated and experimental recordings. Similar findings are obtained with irradiance variations at temperature of 25 °C as shown in Fig. 20a,b. Both figures indicate the high validation of the proposed AHT at different temperatures and solar radiations.

#### Simulated results of scenario 2 for KC200GT PV module

**Figure 21:**
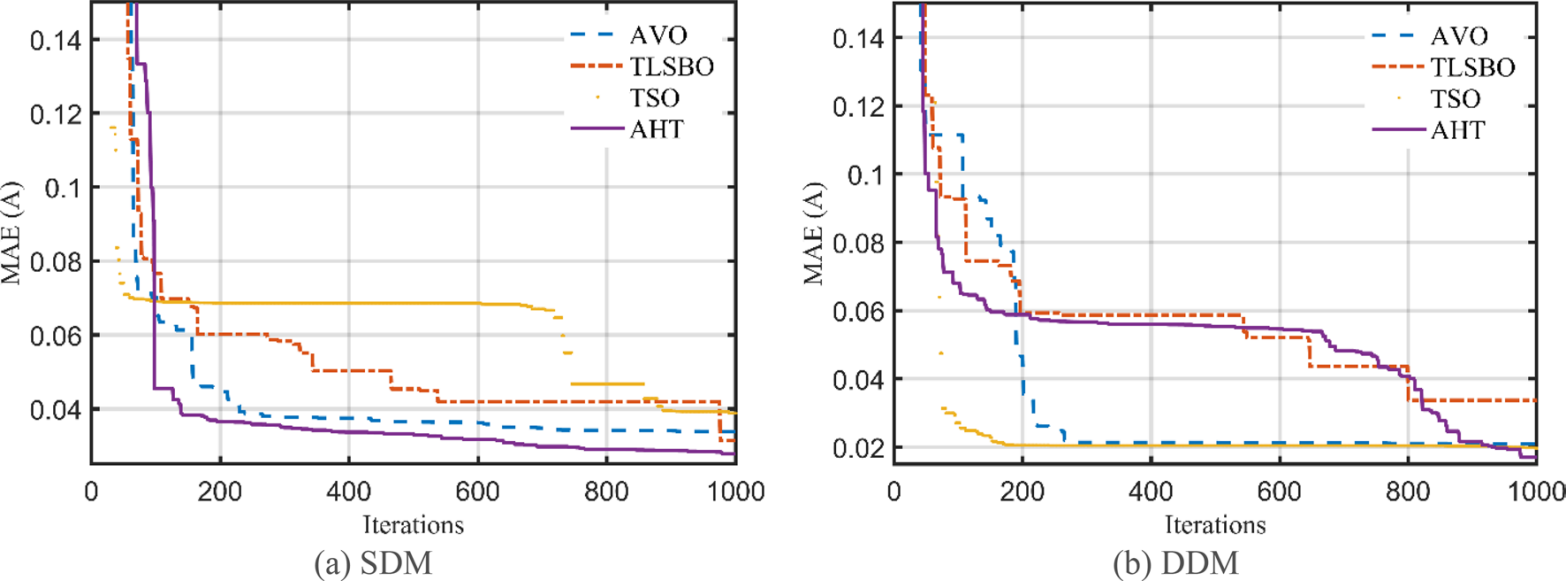
Convergence characteristics of the compared algorithms for the two models of KC200GT (Scenario 2).

For this scenario, the proposed AHT, AVO, TSO, and TLSBO are performed and the regarding parameters of SDM and DDM are depicted in Table 9. Also, the convergence characteristics of the AHT in comparison with AVO, TLSBO, and TSO for this scenario are developed for SDM and DDM as illustrated in Fig. 21a,b. As shown, for SDM, the proposed AHT could achieve the lowest MAE value of 2.79E−2, whilst AVO, TSO, and TLSBO achieve the lowest possible values of 3.39E−2, 3.14E−2 and 3.88E−2, respectively. For the DDM, the AHT could achieve the lowest MAE value of 1.70E−2, whilst AVO, TSO, and TLSBO achieve the lowest possible values of 2.11E−2, 3.37E−2 and 1.97E−2, accordingly. Based on these findings, the proposed AHT declares improvement percentage of 17.75%, 11.30% and 28.15% based on the SDM and 19.24%, 49.59% and 13.55% based on the DDM compared to AVO, TSO, and TLSBO, respectively.

**Table 9:** Extracted parameters by the compared algorithms for KC200GT PV module of Scenario 2 (Results are reported per cell).

Parameter	SDM				DDM			
	AVO	TLSBO	TSO	AHT	AVO	TLSBO	TSO	AHT
$I_{ph}$(A)	8.198778	8.191654	8.229325	8.190629	8.190013	8.196264	8.203874	8.196342
$R_{S}$(Ω)	0.004265	0.004596	0.004282	0.004367	4.68E−03	4.40E−03	4.74E−03	4.70E−03
$R_{Sh}$(Ω)	82.31924	100	7.840211	92.61766	35.64147	25.25724	17.14622	18.72093
$I_{SD1}$(μA)	1.64E−7	7.54E−8	1.34E−7	1.23E−7	4.19E−8	1.10E−7	3.02E−7	1.10E−10
$n_{1}$	1.337388	1.281796	1.322714	1.316375	1.244973	1.308085	1.432184	1.000402
$I_{SD2}$(μA)	–	–	–	–	7.57E−7	0	2.64E−10	1.35E−7
$n_{2}$	–	–	–	–	1.827928	1.706817	1.017269	1.346679
MAE	3.39E−2	3.14E−2	3.88E−2	2.79E−2	2.11E−2	3.37E−2	1.97E−2	1.70E−2

**Figure 22:**
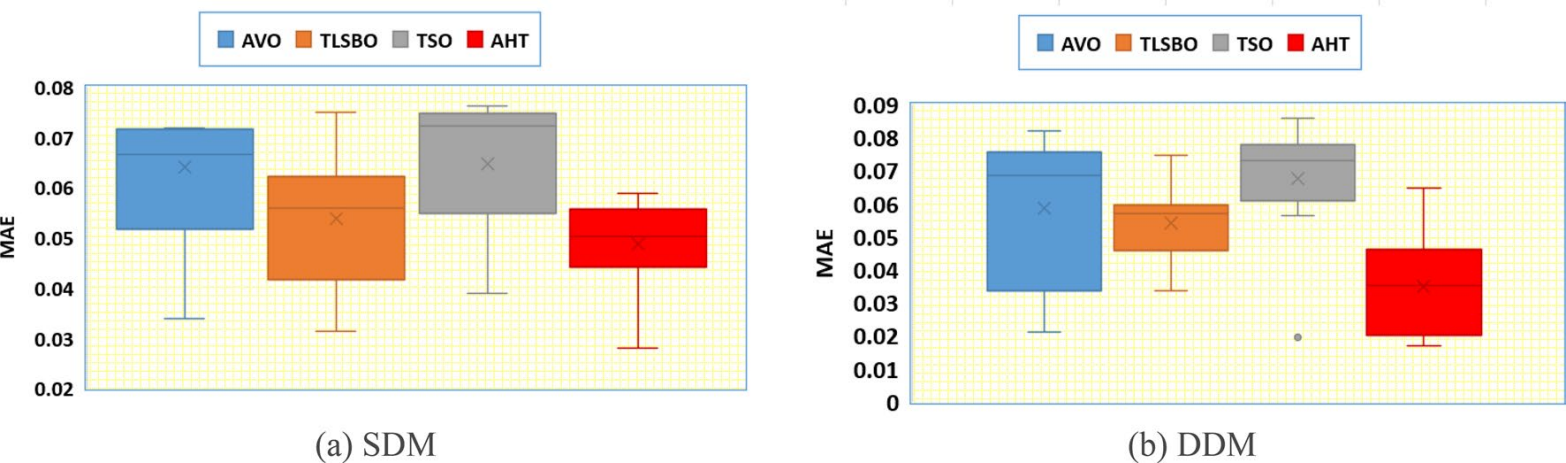
Whisker’s plot of the AHT in comparison with AVO, TLSBO, and TSO with the two models of KC200GT (Scenario 2).

In addition, Fig. 22 displays the regarding Whisker’s plot of the AHT in comparison with AVO, TLSBO, and TSO for this scenario.

As shown, for SDM, AHT derives the least minimum, mean, maximum and standard deviation related to the MAE of 0.0279, 0.0488, 0.0589 and 0.0092, respectively. On the other side, AVO, TLSBO and TSO obtain higher standard deviations of 0.0183, 0.0133 and 0.0136, respectively. Similarly, for the DDM, the proposed AHT shows the best performance with the least minimum, mean and maximum MAE values of 0.017, 0.0349 and 0.0647, respectively.

**Figure 23:**
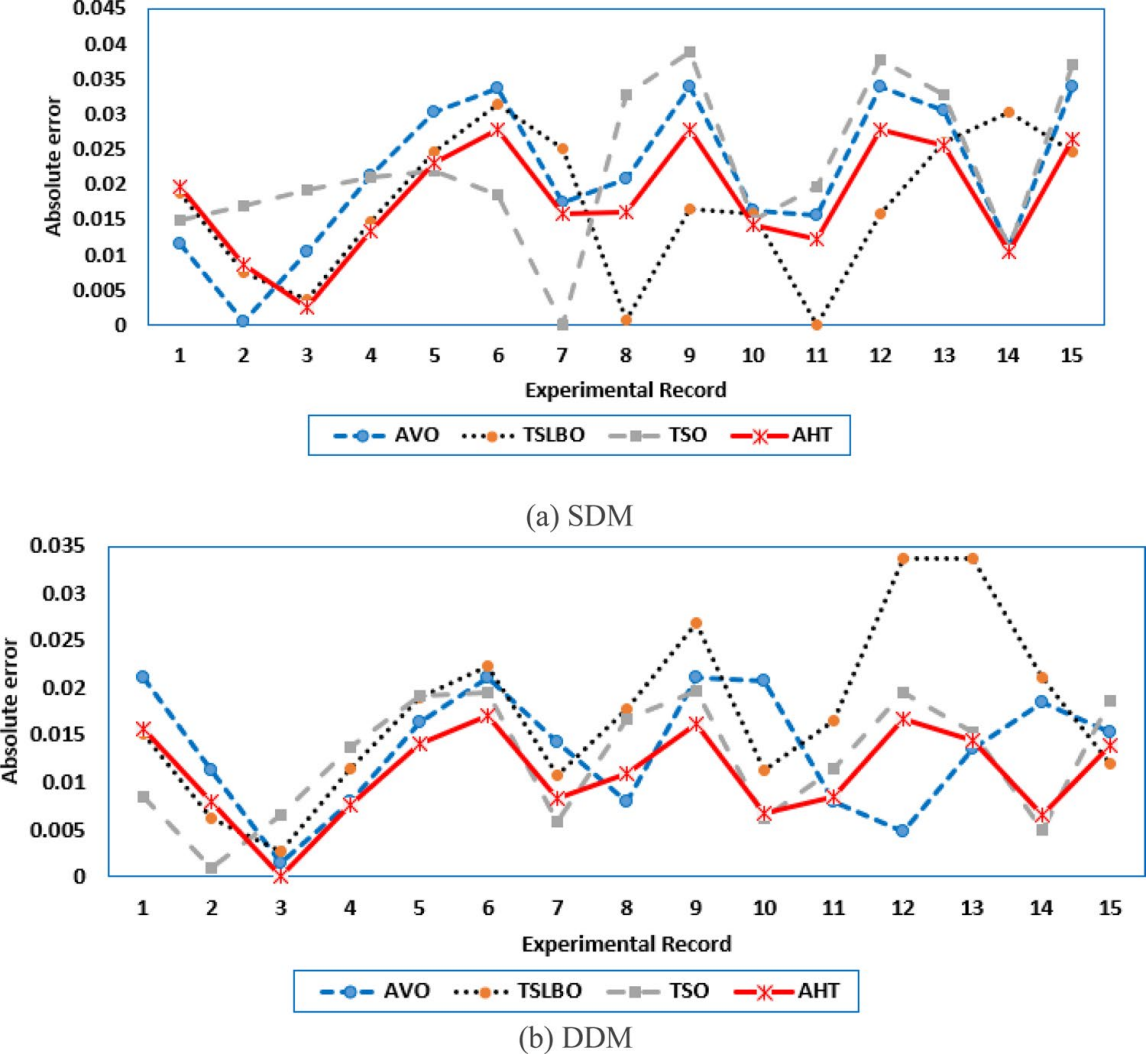
Absolute errors in the produced current by the compared optimizers of KC200GT PV module (Scenario 2).

For both models at this scenario, Fig. 23a,b describe the experimental absolute errors in the produced current for the AHT, AVO, TLSBO, and TSO, respectively. As shown, the proposed AHT derives superior capability with better distribution of errors compared to the others. For the SDM, the errors using the proposed AHT range from 0.0026 at the reading no. 3 to 0.0279 at the reading no. 12. Therefore, the regarding difference between the two obtained boundaries is 0.0253. In similar way, the calculated difference between the two obtained boundaries using AVO, TLSBO, and TSO are 0.0333, 0.0313 and 0.0388, respectively. These differences demonstrate the high capability of the proposed AHT in achieving the best distribution of the errors over the course of the experimental recordings.

For the DDM, the errors using the proposed AHT range from 4.18E−5 at the reading no. 3 to 0.01701 at the reading no. 6. The regarding difference between the two obtained boundaries is 0.001697. In similar way, the calculated difference between the two obtained boundaries using AVO, TLSBO, and TSO are 0.01963, 0.03101 and 0.01874, respectively. These differences demonstrate the high capability of the proposed AHT in achieving the best distribution of the errors over the course of the experimental recordings.

### PHOTO WATT-PWP 201 PV module

#### Simulated results of scenario 1 for PWP 201 PV module

**Figure 24:**
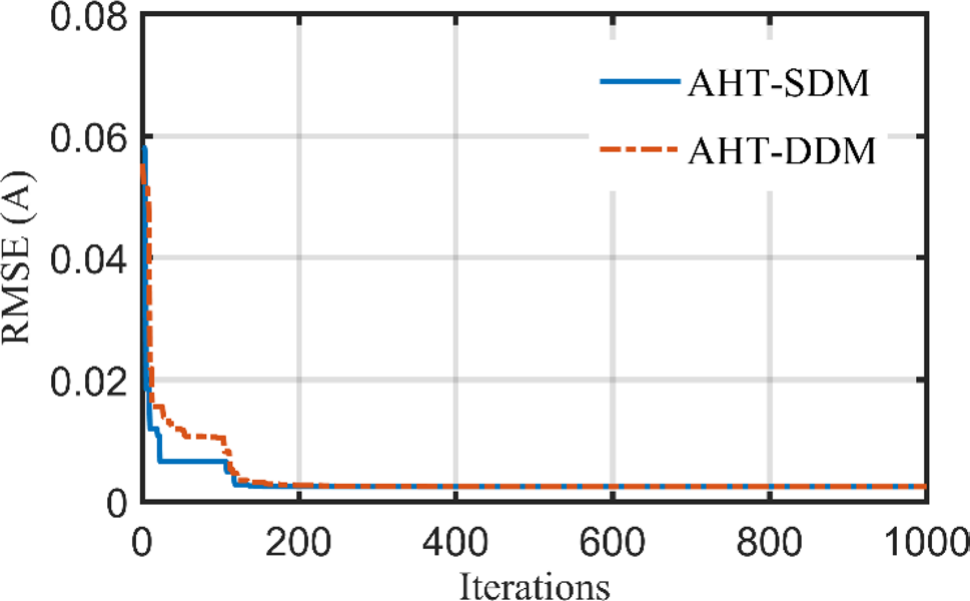
Convergence patterns of AHT with SDM and DDM for PWP 201 polycrystalline PV module (Scenario 1).

**Figure 25:**
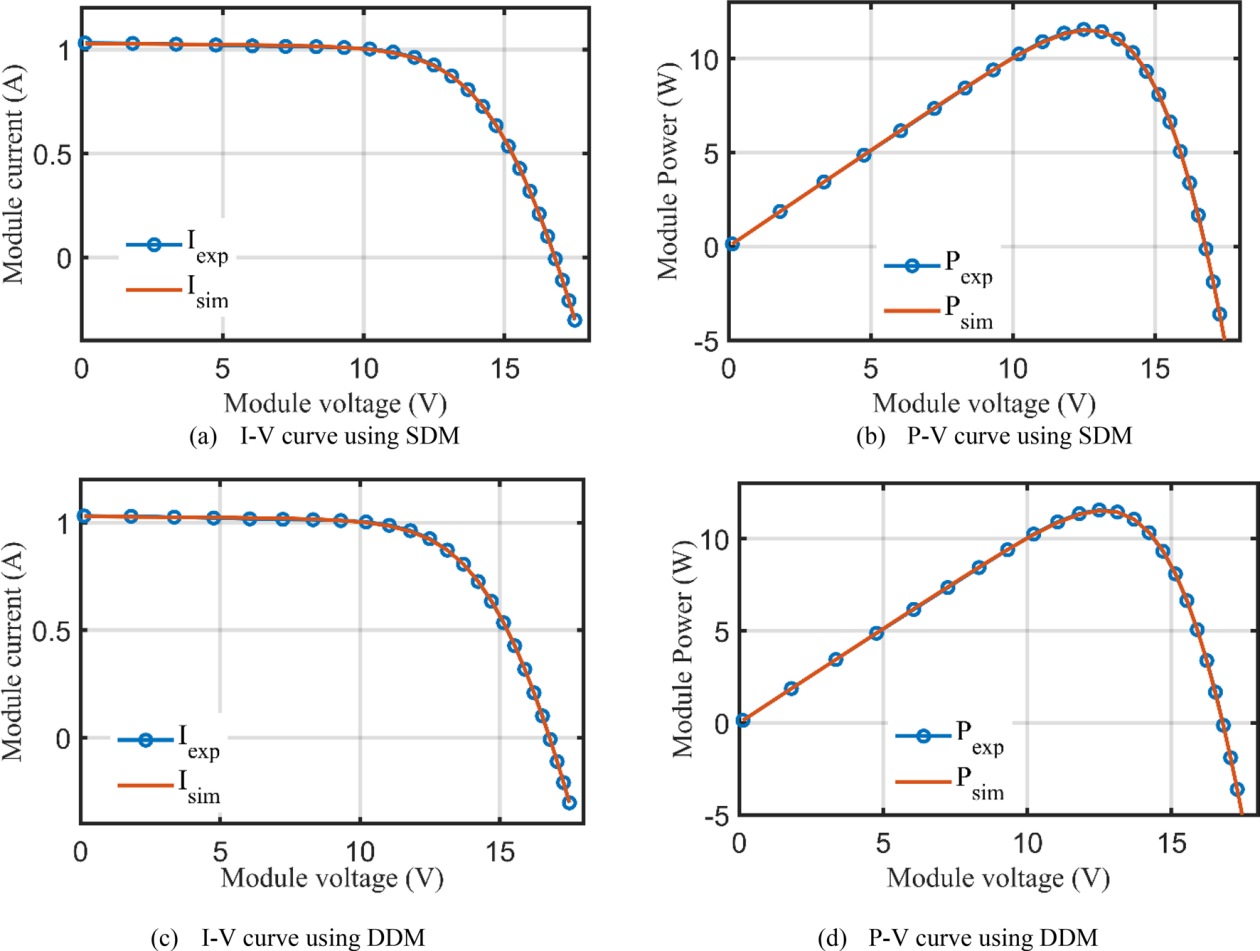
I–V and P–V curves of experimental and simulated results for PWP 201 PV module (Scenario 1).

Table 10 describes the five and seven-nine variables of SDM and DDM, respectively, that were obtained using the AHT considering both scenarios 1 and 2. According to Table 10, for the first scenario, the AHT approach determines that 2.42507 mA is the optimum adaption value for both SDM and DDM. The minimal RMSE associated with each model's parameters is shown in this table. The suggested AHT demonstrates strong stability and great searching efficiency than previously reported algorithms, according to the statistical evaluation of the three metrics (Best, Average, Worst) presented in Table 10. The thirty runs' results demonstrate the suggested AHT’s superior resilience over others as related to the comparison of the proposed AHT and other recently described approaches has been presented in Table 11 which are SNS^72^, FBI^44^, SA^49^, ISCE^53^, ImCSA^52^, HFAPS^69^, SMA^15^, CGBO^73^, PSO^64^, and RAO optimizer^13^ for the SDM. The comparative assessment is conducted considering the DDM versus different reported state of the art such as SNS^72^, FBI^44^, PSO^74^, LAPO^75^, PSO^64^, MPA^73^ and CGBO^73^. Furthermore, Fig. 24 illustrates the AHT’s convergence properties which demonstrates how the suggested AHT shows high performances for both SDM and DDM. Additionally, Fig. 25a–d exhibits the projected and measured values for the powers and currents at each point of the SDM and DDM of this module, characterizing the similarity between the anticipated and measured values while estimating the data with the proposed AHT.

**Table 10:** Extracted parameters based on AHT for PWP 201 PV Module.

Parameters	Scenario 1		Scenario 2	
	SDM	DDM	SDM	DDM
$I_{ph}$ (A)	1.030514	1.030514	1.029358	1.029234
$R_{S}$ (Ω)	0.033369	0.033369	0.03349	0.033324
$R_{Sh}$ (Ω)	27.27729	27.27726	28.39074	29.09406
$I_{SD1}$ (μA)	3.4800E−6	3.4800E−6	3.2800E−6	3.4100E−6
$n_{1}$	1.35119	1.35119	1.345064	1.349066
$I_{SD2}$ (μA)	–	1.9000E−12	–	4.3400E−8
$n_{2}$	–	1.351177	–	1.930731
RMSE (mA)	2.42507	2.42507	2.60800	2.59300
MAE (mA)	4.4320	4.4320	3.6600	3.7110

**Table 11:** Comparative assessment of AHT versus reported algorithms for PWP 201 polycrystalline PV Module (Scenario 1).

Optimizer	SDM		
	Min (RMSE)	Mean (RMSE)	Max (RMSE)
AHT	2.42507E−3	2.42509E−3	2.4253E−3
SNS^72^	2.4350E−3	2.4680E−3	2.5470E−3
FBI^44^	2.4250E−3	2.4260E−3	2.4350E−3
SA^49^	2.617E−3	–	–
ISCE^53^	2.4251E−3	2.4251E−3	2.4251E−3
ImCSA^52^	2.425E−3	2.4251E−3	2.4251E−3
HFAPS^69^	2.4251E−3	–	–
SMA^15^	2.8110E−3	3.3530E−3	1.0799E−2
CGBO^73^	2.425101E−3	–	–
PSO^64^	2.4390E−3	2.3666E−2	9.7700E−2
MPA^73^	2.59141E−3	–	–
RAO^13^	2.8220E−3	3.2960E−3	4.2554E−1

Optimizer	DDM		
	Min (RMSE)	Mean (RMSE)	Max (RMSE)
AHT	2.42507E−3	2.43307E−3	2.49922E−3
SNS^72^	2.4410E−3	2.6020E−3	3.1800E−3
FBI^44^	2.4250E−3	2.431E−3	2.4430E−3
PSO^74^	3.2066E−3	−	−
LAPO^75^	3.2734E−2	4.3132E−2	5.7507E−2
PSO^64^	3.3925E−3	2.0808E−2	3.3742E−2
MPA^73^	2.6227E−3	–	–
CGBO^73^	2.4510E−3	–	–

#### Simulated results of scenario 2 for PWP 201 PV module

**Figure 26:**
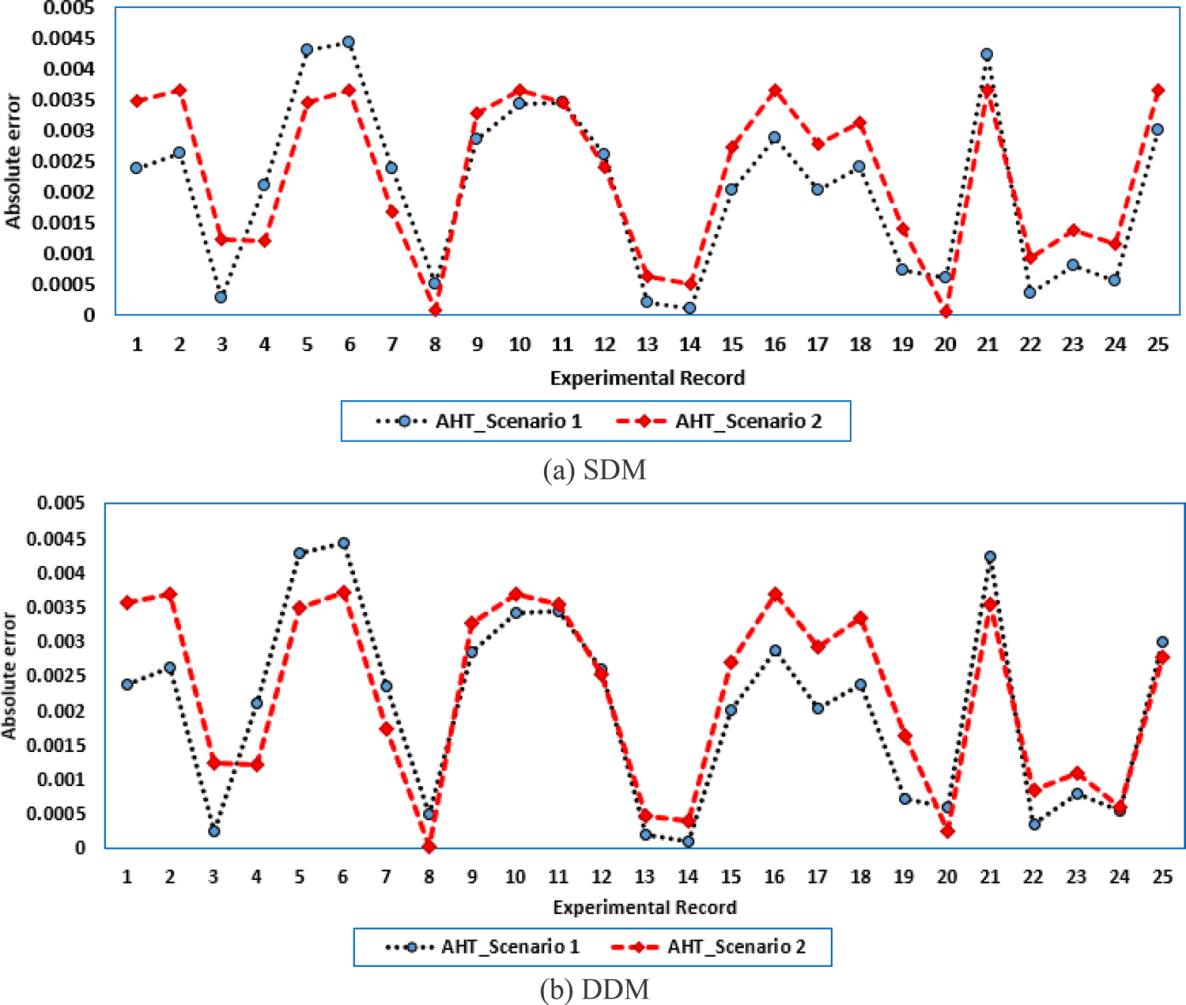
Absolute errors in the produced current by the AHT for both scenarios of PWP 201 PV module.

For this scenario, the proposed AHT is performed and the regarding parameters of SDM and DDM are previously stated in Table 10. For both models and scenarios, Fig. 26a,b display the experimental absolute errors in the produced current using the proposed AHT. For the SDM, in the first scenario, the errors using the proposed AHT range from 9.21E−5 to 4.43E−3 with regarding difference between the two obtained boundaries of 4.34E−3. For the same model, the errors using the proposed AHT range from 4.40E−5 to 3.66E−3 with regarding difference between the two obtained boundaries of 3.62E−3 considering the second scenario. Based on that, the utilization of the MAE minimization objective at Scenario 2 shows better error distribution with 16.67% improvement over the RMSE minimization objective at Scenario 1 via the proposed AHT. Similar findings are attained considering the SDM. The utilization of the MAE minimization objective at Scenario 2 shows better error distribution with 15.15% improvement over the RMSE minimization objective at Scenario 1 via the proposed AHT. In the first scenario, the errors using the proposed AHT range from 9.22E−5 to 4.43E−3 with regarding difference between the two obtained boundaries of 4.34E−3. For the same model, the errors using the proposed AHT range from 2.9E−5 to 3.71–3 with regarding difference between the two obtained boundaries of 3.68E−3 considering the second scenario.

**Figure 27:**
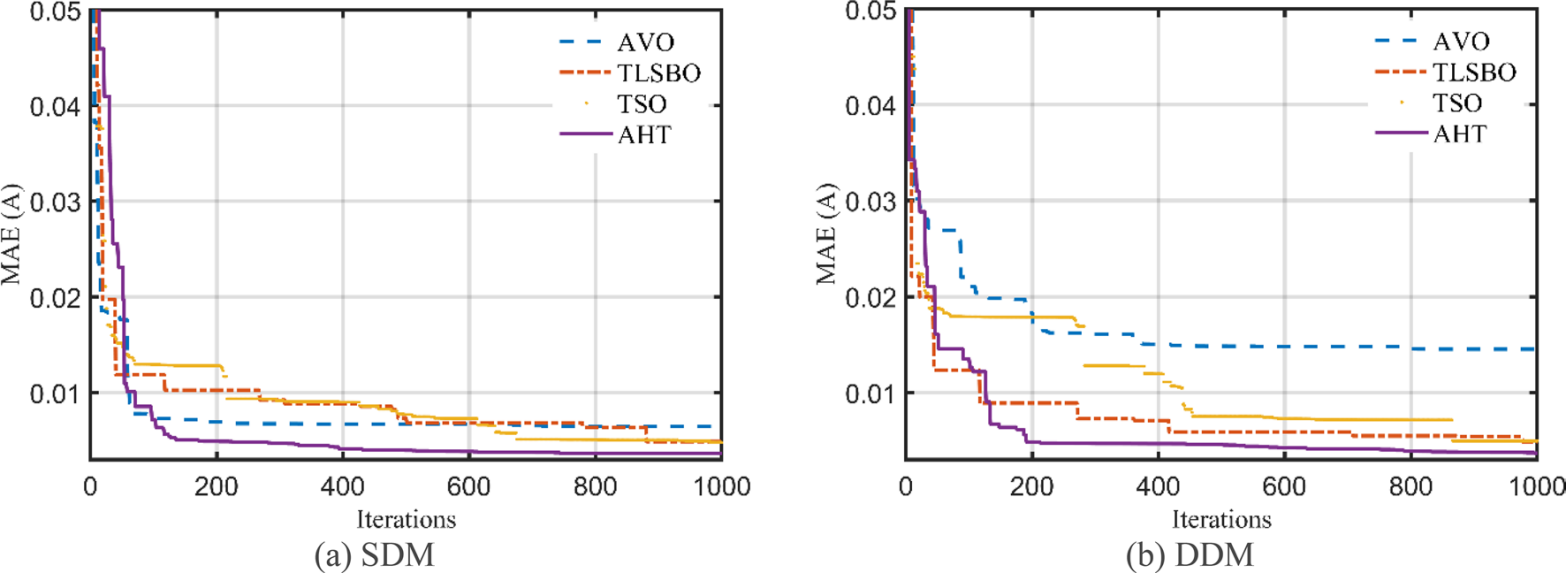
Convergence characteristics of the compared algorithms for the two models of PWP 201 (Scenario 2).

Moreover, a comparative assessment between the proposed AHT, AVO, TSO, and TLSBO is performed considering this scenario. The regarding parameters of SDM and DDM are depicted in Table 12 while their convergence characteristics are developed for SDM and DDM as illustrated in Fig. 27a,b. As shown, the proposed AHT could achieve the lowest MAE value of 3.66E−3 and 3.71E−3, for SDM and DDM, respectively.

**Table 12:** Extracted parameters by the compared algorithms for PWP 201 PV module of Scenario 2 (Results are reported per cell).

Parameter	SDM				DDM			
	AVO	TLSBO	TSO	AHT	AVO	TLSBO	TSO	AHT
$I_{ph}$ (A)	1.038252	1.027478	1.02759	1.029358	1.038579	1.027355	1.028935	1.029234
$R_{S}$ (Ω)	0.035851	0.031499	0.033269	0.03349	0.02283	0.031888	0.031795	0.033324
$R_{Sh}$ (Ω)	11.79168	48.77022	42.25134	28.39074	55.10929	50.10676	37.70859	29.09406
$I_{SD1}$ (μA)	1.2700E−6	5.7000E−6	3.5400E−6	3.2800E−6	1.61E−5	8.3700E−7	0	3.4100E−6
$n_{1}$	1.252037	1.40606	1.352615	1.345064	1.622192	1.996522	1.997618	1.349066
(μA)	–	–	–	–	2.62E−5	5.4000E−6	5.07E−6	4.3400E−8
$n_{2}$	–	–	–	–	1.745288	1.400507	1.392475	1.930731
MAE	6.49E−3	4.90E−3	4.81E−3	3.66E−3	1.45E−2	4.88E−3	4.97E−3	3.71E−3

**Figure 28:**
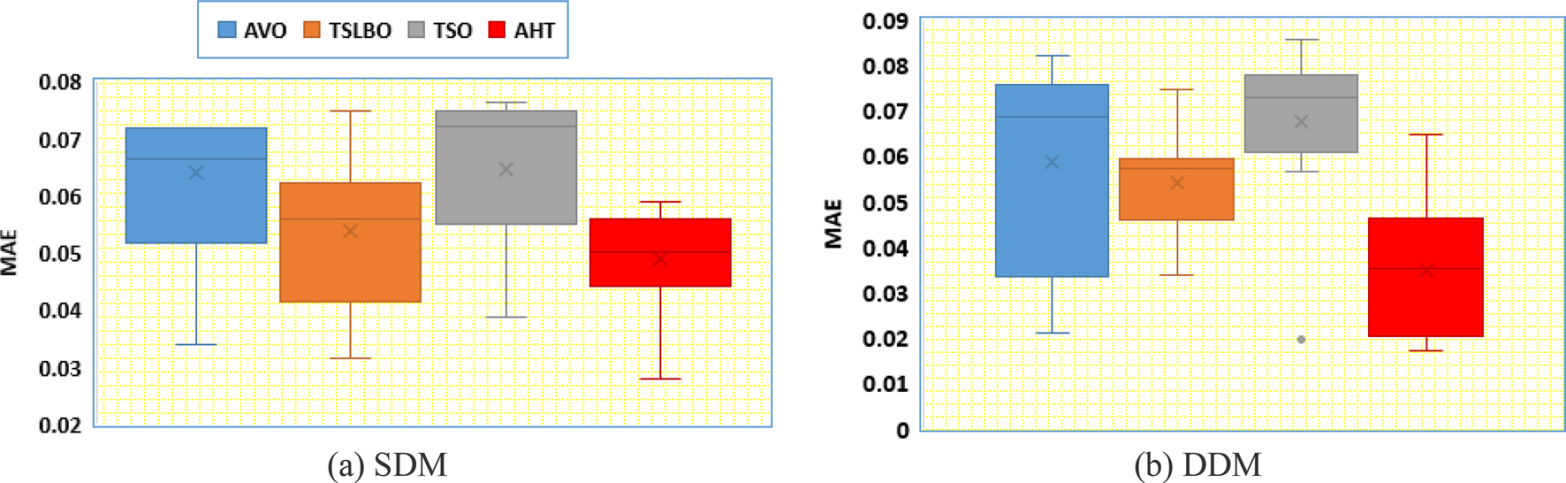
Whisker’s plot of the AHT in comparison with AVO, TLSBO, and TSO with the two models of PWP 201 (Scenario 2).

In addition, Fig. 28 displays the regarding Whisker’s plot of the AHT in comparison with AVO, TLSBO, and TSO for this scenario. As shown, the AHT shows the best performance compared to the others. The proposed AHT derives the least minimum, mean, and maximum related to the MAE of 0.00366, 0.00451 and 0.00628 for the SDM and 0.00371, 0.005696 and 0.01286 for the DDM, respectively.

## Conclusion

This study has presented a novel application of an Artificial Hummingbird Technique (AHT) for extracting the unknown parameters from SDM and DDM PV models of mono-crystalline STM6-40/36, and multi-crystalline KC200GT. The performance of the proposed AHT is assessed by statical indices called Min RMSE, Max RMSE, Mean RMSE, Standard deviation, IAE, PAE, P–V and I–V curves. The earlier results of AHT for determining accurate parameters of various PV models illustrate that AHT produces a competitive end-result versus other recently developed algorithms. The parameters of the PV module are extracted using the AHT in this article. To estimate the PV module parameters, the proposed approach uses experimental data extracted from the Power–Voltage (P–V) curve. At a final stage of this effort, Photo WATT-PWP 201 has been examined. To sum up, three distinct PV modules, which are widely used in the literature namely, STM6-40/36, KC200GT and Photo WATT-PWP 201 have been investigated to validate the proposed AHT. For all PV modules, the proposed AHT exhibits the lowest RMSE. The performance of the AHT is additionally tested utilizing statistical data overall 30 independent runs. Based on the experimental results, it may be announced that the AHT overcomes all the selected state-of-the-art optimizers for the reported test cases.

## Data Availability

The data that support the findings of this study are available from the corresponding author upon reasonable request.
